# Visualizing Arc protein dynamics and localization in the mammalian brain using AAV-mediated *in situ* gene labeling

**DOI:** 10.3389/fnmol.2023.1140785

**Published:** 2023-06-15

**Authors:** Martino Avallone, Joaquín Pardo, Tadiwos F. Mergiya, Jana Rájová, Atte Räsänen, Marcus Davidsson, Malin Åkerblom, Luis Quintino, Darshan Kumar, Clive R. Bramham, Tomas Björklund

**Affiliations:** ^1^Molecular Neuromodulation, Wallenberg Neuroscience Center, Lund University, Lund, Sweden; ^2^Instituto de Investigaciones Bioquímicas de La Plata “Prof. Dr. Rodolfo R. Brenner” (INIBIOLP), Consejo Nacional de Investigaciones Científicas y Técnicas (CONICET)—Universidad Nacional de La Plata (UNLP), La Plata, Argentina; ^3^Department of Biomedicine, University of Bergen, Bergen, Norway; ^4^Mohn Research Center for the Brain, University of Bergen, Bergen, Norway; ^5^CNS Gene Therapy, Department of Experimental Medical Sciences, Lund University, Lund, Sweden; ^6^Aiforia Technologies Oyj, Helsinki, Finland

**Keywords:** retrotransposon, synaptic plasticity, AAV, PLA, HITI, cell–cell transfer

## Abstract

The activity-regulated cytoskeleton-associated (Arc) protein is essential for synaptic plasticity and memory formation. The Arc gene, which contains remnants of a structural GAG retrotransposon sequence, produces a protein that self-assembles into capsid-like structures harboring Arc mRNA. Arc capsids, released from neurons, have been proposed as a novel intercellular mechanism for mRNA transmission. Nevertheless, evidence for intercellular transport of Arc in the mammalian brain is still lacking. To enable the tracking of Arc molecules from individual neurons *in vivo*, we devised an adeno-associated virus (AAV) mediated approach to tag the N-terminal of the mouse Arc protein with a fluorescent reporter using CRISPR/Cas9 homologous independent targeted integration (HITI). We show that a sequence coding for mCherry can successfully be knocked in at the 5′ end of the Arc open reading frame. While nine spCas9 gene editing sites surround the Arc start codon, the accuracy of the editing was highly sequence-dependent, with only a single target resulting in an in-frame reporter integration. When inducing long-term potentiation (LTP) in the hippocampus, we observed an increase of Arc protein highly correlated with an increase in fluorescent intensity and the number of mCherry-positive cells. By proximity ligation assay (PLA), we demonstrated that the mCherry-Arc fusion protein retains the Arc function by interacting with the transmembrane protein stargazin in postsynaptic spines. Finally, we recorded mCherry-Arc interaction with presynaptic protein Bassoon in mCherry-negative surrounding neurons at close proximity to mCherry-positive spines of edited neurons. This is the first study to provide support for inter-neuronal *in vivo* transfer of Arc in the mammalian brain.

## Introduction

Cell-to-cell communication between neurons in the mammalian brain is primarily restricted to chemical or electrical synapses. Other mechanisms of communication have also been proposed, such as exosomes (Men et al., 2019; Pascual et al., 2020; Vilcaes et al., 2021; Xia et al., 2022) and tunneling nanotubes (Tardivel et al., 2016; Kalargyrou et al., 2021; Khattar et al., 2022). A recent non-canonical carrier of information is proposed to be secreted viral-like particles (VLPs). This idea originates from studies observing that the activity-regulated cytoskeleton-associated protein (Arc) can assemble into capsid-like structures which can harbor mRNA molecules within (Ashley et al., 2018; Pastuzyn et al., 2018; Eriksen et al., 2021; Hantak et al., 2021; Eriksen and Bramham, 2022).

Two studies published back-to-back observed that these VLPs can be taken up by cultured primary mouse neurons (Pastuzyn et al., 2018) and transferred across the neuromuscular synapse in *Drosophila* larvae, respectively (Ashley et al., 2018). The Arc gene is postulated to be an ancient remnant of a retroviral infection with essential components of a GAG protein intact (Campillos et al., 2006; Zhang et al., 2015; Shepherd, 2018). The studies further demonstrate that this GAG protein can self-assemble into retrovirus-like capsids. Taken together, these observations raise the intriguing possibility that there may be a VLP-mediated cell-to-cell communication in the brain, which could transfer mRNA between cells. However, such transfer has not yet been shown in the mammalian brain, and its potential function is unknown. Consequently, methods are urgently needed to study Arc protein localization and transfer between neurons *in vivo*.

Sparsely labeling the Arc gene *in situ* in the mammalian brain would allow for visualization and tracking of the protein by fluorescence microscopy. One successful approach to enable knocking-in tags into expressed genes in non-dividing cells *in situ* is based on the clustered regularly interspaced short palindromic repeat (CRISPR)/Cas9 homologous independent targeted integration (HITI) (Suzuki et al., 2016). Several versions have been developed from this system, providing additional tools for editing in the mammalian brain. The HiUGE (homology-independent universal genome engineering) (Gao et al., 2019) and the Orange (open resource for the application of neuronal genome editing) (Willems et al., 2020) have both been used to knock in protein-coding sequences at multiple loci in postmitotic neurons *in vivo*. A hybrid version of the HITI system, named SATI (intercellular linearized Single homology Arm donor mediated intron-Targeting Integration), which fuses NHEJ (non-homologous end joining) and HDR (homology-directed repair), was developed as an improvement to HITI (Suzuki et al., 2019). Another system called vSLENDR (virus-mediated single-cell labeling of endogenous proteins *via* HDR), was solely based on HDR and showed high gene editing (Nishiyama et al., 2017). However, concerns have been raised about potential background expression (Nishizono et al., 2020).

**Figure 1:**
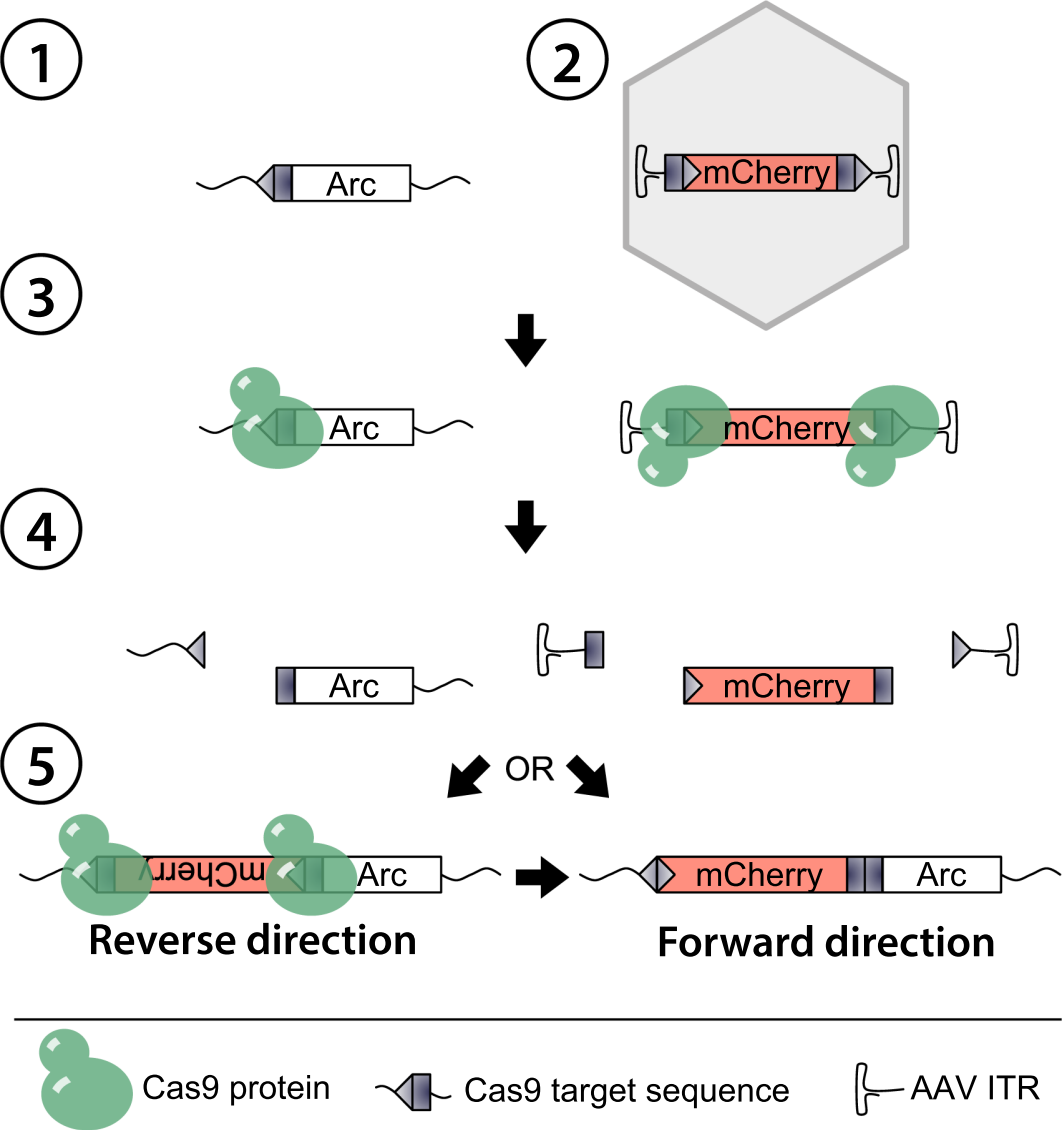
Schematic of HITI-mediated gene editing in Arc. The genomic Arc locus is here the target ①. An AAV containing a tailored donor template (DT) mCherry ② co-expressed with a single guide RNA is delivered together with Cas9 through direct injection. As Cas9 expresses, the nuclease and the gRNA assemble and target the matching sequences in the Arc gene and the flanking regions of the DT ③. Subsequently, a double-strand break occurs ④, and the NHEJ repair mechanism initiates in the nucleus to repair the Arc gene. The available exogenous DNA is inserted during the NHEJ either in a reverse or forward direction ⑤. In the first case, the targeted sequence is rebuilt, enabling a re-cut by Cas9, while the DT is irreversibly knocked-in in the latter.

As sparse labeling of neurons is required to study individual cells, we chose adeno-associated virus (AAV)-mediated CRISPR/Cas9 HITI for the knock-in of a fluorescent marker into the N-terminal region of the Arc protein (Figure 1). We constructed an AAV system delivering Cas9 and a reporter gene (mCherry) together with a single guide RNA (sgRNA). To optimize the efficacy, we targeted several putative spCas9 amenable genomic sites surrounding the start codon of the Arc open reading frame (ORF) (Supplementary Figure S1A). We injected our AAV system into the mouse striatum and hippocampus and visualized the Arc protein using immunohistochemistry and proximity ligation assay (PLA). We assessed the expression under baseline conditions and after long-term potentiation (LTP) induction. In this work, we show that it is possible to knock-in a fluorescent reporter into the Arc ORF by CRISPR/Cas9 HITI in the mouse brain. This approach allows the production of a chimeric functional Arc protein in a sparse population of transduced neurons, thus paving the way to perform *in vivo* synapse and cell-to-cell communication studies of Arc in the intact brain.

## Materials and methods

### Evaluation of CRISPR/Cas9 target sequences

A stretch of 90 bp in the C57bl/6 Arc locus (51 bp upstream and 39 bp downstream of the Arc start codon) was analyzed using gRNAs web-based evaluation tools. The Cas9 system adopted from the *Streptococcus pyogenes* restricts the identification of gRNAs by the PAM sequence NGG, identifying 11 possible insertion sites (Supplementary sgRNAs Table). Among them, two did not fit the HITI system since repairing the DSB with the donor template (DT) would have generated an in-frame stop between the inserted sequence and the Arc gene. One putative gRNA was located downstream of an endogenous stop codon in frame with the Arc start codon (TAG-33 bp). In the first experiment, we focused on the possible insertion sites in the 5′ untranslated region (UTR) and skipped those inside the ORF. Consequently, we had four guides that could potentially be used. The screening tool we used to select the best candidates was from the CCTop—CRISPR/Cas9 target online predictor (Stemmer et al., 2015; Labuhn et al., 2018). However, we did monitor these guides on two more websites for extra insights: CRISPR-Cas9 guide RNA design checker (IDT, 2022) and “CHOP-CHOP” (Montague et al., 2014; Labun et al., 2016, 2019). This resulted in the selection of sg2[−] and sg4[−] for the first experiment.

**Figure 2:**
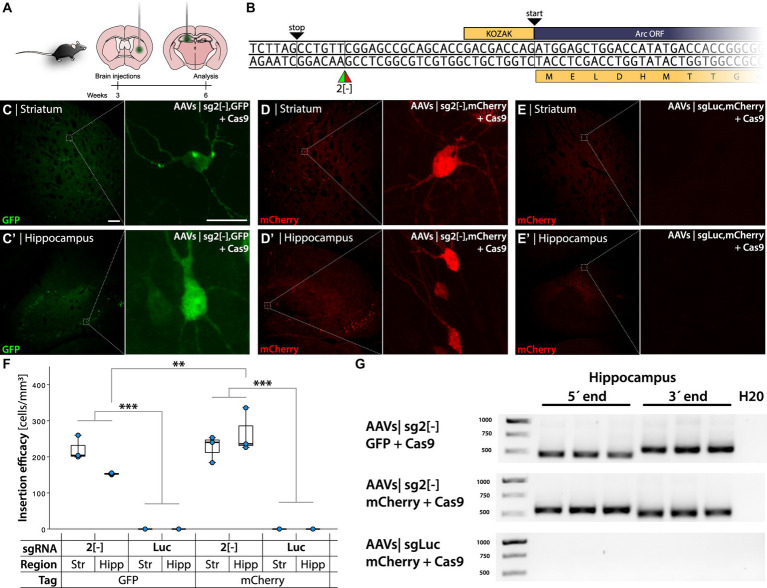
HITI-mediated gene editing when targeting the Arc 5′ UTR [−] strand. **(A)** Schema of AAVs injections in the striatum and hippocampus of C57bl/6 mice, with time course of the experimental design. **(B)** 5′ Arc sequence. TAG stop codon in frame with the ATG start codon of the Arc gene. 2[−] is the target site of the gRNA designed for the insertion of the GFP and mCherry DT. **(C–E′)**. Confocal images from the striatum and hippocampus after IHC. **(C,D)** Refer to GFP and mCherry knock-in, showing green and red fluorescent cells, respectively. **(E)** Control group. In **(C)** the left scale bar is 50 μm, and in the right, it is 20 μm. **(F)** Quantification of cellular knock-in efficiency showing cell density in the experimental groups. ^*^Statistically different (one way ANOVA *p* ≤ 0.05, followed by Tukey’s HSD) (*n* = 3). **(G)** PCR amplicons on bulk DNA extracted from the hippocampus, amplifying the 5′ and 3′ regions of the knocked-in DT.

**Figure 3:**
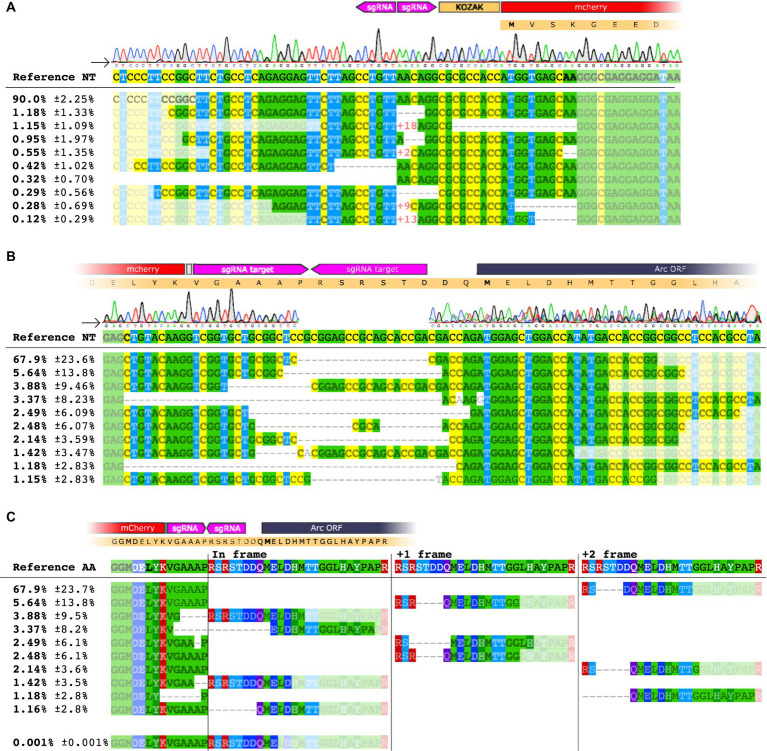
NGS analysis of HITI targeting the Arc 5′ UTR [−] strand. **(A)** Sequence alignment on the 5′ region of the knocked-in mCherry insert at the 2[−] site. The upper panel shows base nucleotide calls from Sanger sequencing. The lower panel displays NGS analysis with percentages of the 10 most common amplicons. **(B)** Sequence alignment for the mCherry insert conducted on the 3′ region. **(C)** Amino acid sequence of the 3′ end region. Each amplicon is depicted showing the frameshift resulting from the deletion between the inserted sequence and the Arc gene.

Due to the findings in the first experiment (Figure 2; Supplementary Figure S2), we focused on the [+] DNA strand target sites for the second experiment and therefore selected sg1[+] and sg9[+] (Figure 3).

### Cloning for selected plasmids

Plasmid PX552 (#60958) from Addgene was used as a backbone (Swiech et al., 2015). The first step was to insert each gRNA sequence into the plasmid backbone ITR-U6-sgRNA (backbone)-hSyn-EGFP-KASH-hGHpA-ITR using SapI restriction enzyme (ER1931). The different gRNA oligos were then inserted through ligation into the backbone using T4 ligase. The second step was to insert the DNA fragment containing the tagging sequence flanked by the gRNA-targeted sequences using NEBuilder HiFi DNA Master Mix HiFi (E2621L).

Primers with specific overhangs were used to amplify the donor template sequence using PCR (Supplementary Primer Table). Their products were run in a 1% agarose gel and then gel-extracted using Gel DNA Recovery kit (Zymo Research). A 20 µL Gibson Assembly reaction (NEB) with a total of 0.2 pmol DNA fragment concentration was performed to assemble each fragment into the final plasmid. A 1:2 molar ratio of backbone to insert was used. For mCherry and mEGFP fragments, 140 ng of the vector fragment with 13 ng of the insert fragment were used in the reaction mix, whereas for c-myc 210 ng of the backbone fragment was assembled into 10 or 14 ng of the insert fragment. The reaction was incubated at 50°C for 1 h, purified using DNA Clean & Concentrator (Zymo Research), and then resuspended in 6 μL of elution buffer.

Next, 1 μL of purified Gibson assembly product was transformed into 25 μL of SURE2 Super competent cells (Agilent Technologies) according to the manufacturer’s protocol. Four colonies were selected and grown in 4 mL LB media overnight at 32°C. The DNA was purified using Plasmid Miniprep Kit (Zymo Research) and validated *via* Sanger sequencing (Eurofins Genomics).

All the plasmid constructs produced were made using the same cloning strategy. The control plasmid constructs sgLuc, mCherry used in the first and second experiments (reported in Figures 2, 3, respectively) differ by design. In the first experiment, the sgRNA/Cas9 complex targeted a luciferase sequence which is absent in the mammalian genome, and therefore, no DSBs occurred. In the second experiment, the sgRNA/Cas9 complex again targeted the luciferase sequence, which this time was repeated twice, flanking the DT. In this case, the DT was free to be inserted, but no DBS in the genome allowed the insertion.

### AAV production

HEK 293T cells were cultured in T175 flasks using Dulbecco Modified Eagle Medium (DMEM), 10% fetal bovine serum (FBS), and 1% Penicillin–Streptomycin (P/S) until they reached 80% confluency. Before transfection, the medium was changed with 27 mL of fresh DMEM, FBS, and P/S. A three-plasmid transfection with polyethylenimine (PEI) was carried out using pHGT-1 adenoviral helper plasmid, the engineered MNM008 capsid (modified from AAV2) (Davidsson et al., 2019), and transfer vector in a 1:1:1.2 ratio as described previously (Negrini et al., 2020). Plasmids and PEI were mixed in 3 mL DMEM, incubated at room temperature for 15 min, and added to the cells. Following 24 h after transfection, 27 mL of the medium was replaced with an equal volume of OptiPRO serum-free medium (Thermo Fisher Scientific), 1% P/S. AAVs were harvested from HEK 293T cells 72 h after transfection. First, polyethylene glycol 8000 (PEG8000) precipitation and chloroform extraction led to AAVs extraction. Second, a series of centrifugation in Amicon Ultra-0.5 centrifugal filters (Merck Millipore) purified and concentrated the virus in PBS, which was subsequently stored in glass vials at 4°C (Negrini et al., 2020). Purified AAVs were titrated using ddPCR with primers specific for the ITRs (Lock et al., 2014). For information regarding virus titers, see Table 1. All viruses were normalized to the lowest titer 1.0 × 10^13^ GC/mL (Cas9).

**Table 1:** Titers from the virus batches.

Virus	Titer (GC/mL)
AAVs|sg2[−], GFP	4.3 × 10^13^
AAVs|sgLuc, GFP	5.5 × 10^13^
AAVs|sg2[−], mCherry	2.0 × 10^14^
AAVs|sgLuc, mCherry	6.1 × 10^13^
AAVs|sg1[+], mCherry	1.3 × 10^13^
AAVs|sgLuc, mCherry (exp. 1)	1.5 × 10^13^
AAVs|sg9[+], mCherry	1.4 × 10^13^
AAVs|sgLuc, mCherry (exp. 2)	1.0 × 10^13^
AAVs|sg2[−], mCherry, SATI	1.4 × 10^13^
AAVs|cas9	1.0 × 10^13^

For experiments included in Figure 2, a stoichiometric ratio of 1:1 sgRNA-DT and Cas9 was used. For experiments included in Figure 3 and onward, a stoichiometric ratio of 1:2 was used for each sgRNA-DT and the Cas9.

### Animal research

Female and male C57bl/6 J mice (20 g, 8–9 weeks, Charles River, Germany) were used. All animals were housed with *ad libitum* access to food and water under a 12 h light/dark cycle.

**Figure 4:**
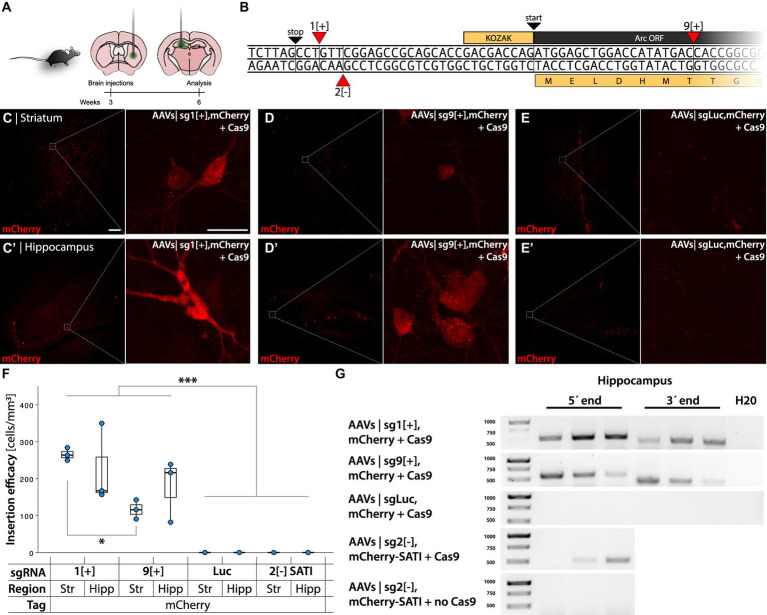
Gene editing targeting the Arc gene with HITI and SATI. **(A)** Schema of AAVs injections in the striatum and hippocampus of C57bl/6 mice. **(B)** 5′ Arc sequence. 1[+] and 9[+] are the insertion sites of the gRNA designed for mCherry insertion employing the HITI system. 2[−] is the insertion site of the gRNA designed for mCherry using the SATI system. **(C–E′)** Confocal images from the striatum and hippocampus after IHC. **(C,D)** Show mCherry+ cells in the active knock-in groups, whereas **(E)** shows the control. In **(C)** the left scale bar is 50 μm, and in the right, it is 20 μm. **(F)** Quantification of knock-in efficiency displayed as fluorescent cell densities. ^*^Statistically different (one way ANOVA, *p* ≤ 0.05, followed by Tukey’s HSD) (*n* = 3). **(G)** PCR amplicons on bulk DNA extracted from the hippocampus, amplifying the 5′ and 3′ regions of the knocked-in DT.

**Figure 5:**
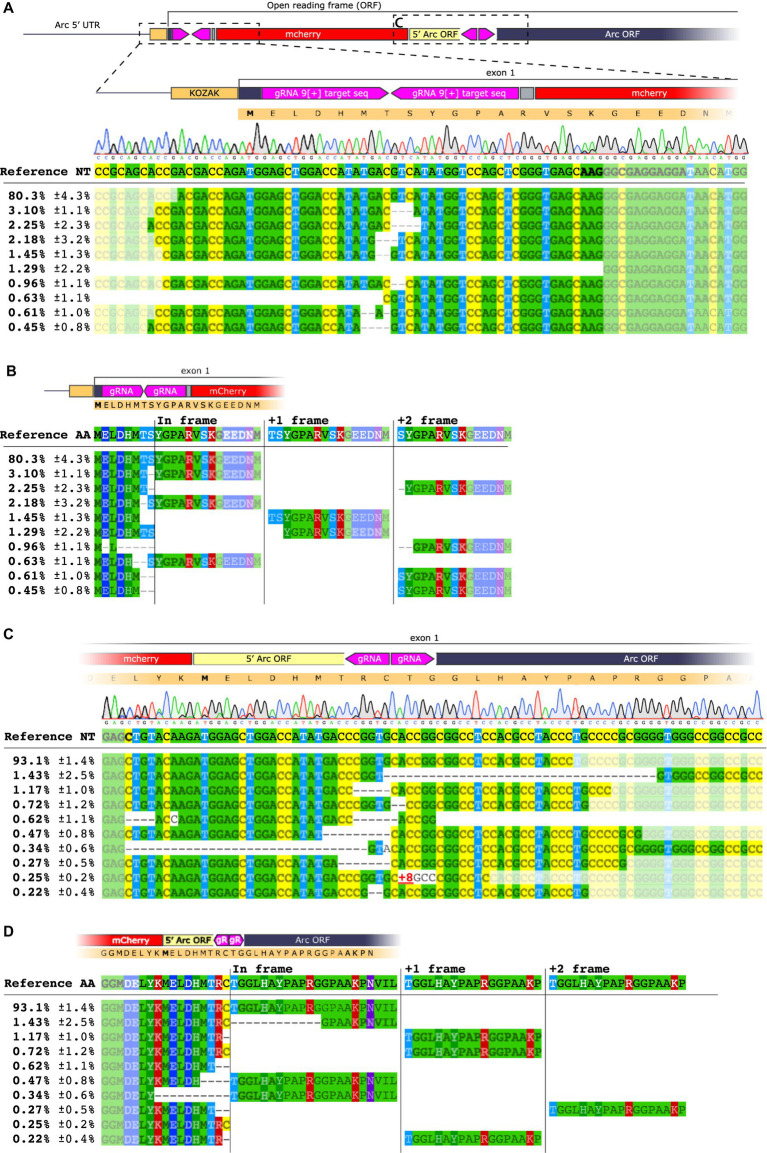
NGS analysis of HITI sg9[+]. **(A)** Sequence alignment on the 5′ region of the knocked-in mCherry insert at the 9[+] site. The upper panel shows base nucleotide calls from Sanger sequencing. The lower panel displays NGS analysis with percentages of the 10 most common amplicons. **(B)** Amino acid sequence of the 5′ end region. Each amplicon is depicted showing the frameshift resulting from the deletion between the inserted sequence and the Arc gene. **(C)** Sequence alignment for the mCherry insert conducted on the 3′ region. **(D)** Amino acid sequence of the 3′ end region.

The experiments behind Figures 2–5 were performed at Lund University, Sweden under the Swedish Animal Welfare Agency regulations and in agreement with the local ethical committee for the use of laboratory animals (Ethical permit no. M 66-16 and 4111/2021-m).

Intrahippocampal AAV injections followed by *in vivo* electrophysiological experiments on long-term potentiation were performed at the University of Bergen. These experimental procedures were approved by Norwegian National Research Ethics Committee in compliance with EU Directive 2010/63/EU, ARRIVE guidelines. Experiments were conducted by the Federation of Laboratory and Animal Science Associations (FELASA) C course-trained and certified researchers.

### Stereotaxic injection

During the surgeries, mice were kept under anesthesia with a 1.2% isoflurane/O_2_ + NO_2_ mixture. The dorsoventral position at ±2.0 mm from the bregma (both rostrocaudal and laterally) was measured to ensure a flat skull position. The coordinates for all injection sites were identified relative to bregma, with the dorsoventral coordinate determined relative to the dura mater. A small hole was drilled in the skull, and each volume was infused in the targeted site using a 5 μL Hamilton syringe fitted with a pulled capillary glass needle. The infusions were carried at a rate of 0.2 μL/min rate, followed by 5 min with the needle left in place to let the volume diffuse. The total volume injected per deposit was 3 μL in the striatum and 2 μL in the hippocampus. The wound was closed with a suture, and the animals were maintained in quarantine under daily observation for 48 h (see Table 2).

**Table 2:** Coordinates were taken relative to bregma.

	Anteroposterior	Mediolateral	Dorsoventral
Str	0.3	1.9	−3.5/−3.2
Hipp	−2	1.5	−2/−1.5

Values are expressed in mm. striatum (Str), hippocampus (Hipp).

### *In vivo* electrophysiology and LTP induction

Long-term potentiation (LTP) induction in the dentate gyrus (DG) was performed 3 weeks after virus injection following previous protocols (Panja et al., 2014). Virus-injected mice (12 weeks old) were anesthetized with urethane (1.2 g/kg, intraperitoneal) and then placed on a stereotaxic frame with a maintained body temperature of 37°C. Two screws (AM system #7935 in stainless steel machine screws, slotted fillister head) placed anterior to bregma on each side of the medial line and touching the cortical surface served as ground and reference electrodes.

**Figure 6:**
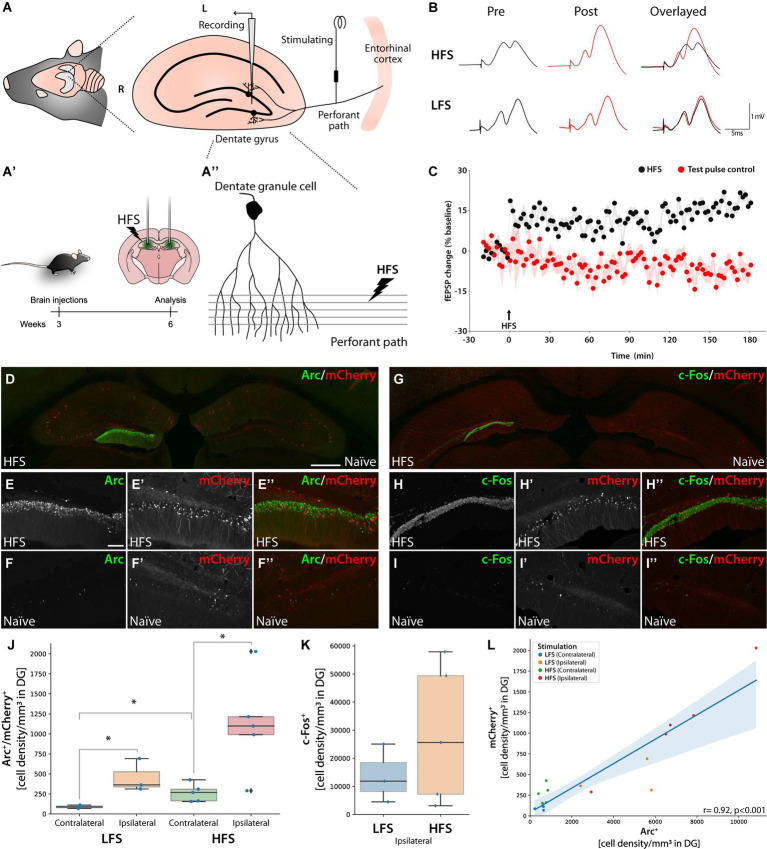
Knocked-in mCherry as an activity reporter gene in dentate granule cell neurons following HFS-induced LTP. Panel **(A)** Illustration of electrode placements for recording perforant path-evoked field potentials in the DG. **(A′)** Schematic illustration of bilateral AAV injections into the DG using AAV| sg9[+], mCherry and Cas9 with time course. Three weeks after HFS performed on the left hippocampus. **(A″)** The stimulating electrode on the perforant path projection from the entorhinal cortex, innervating dendritic arbors of granule cells in the DG. (R: rostral, L: lateral, M: medial, C: caudal). **(B)** Sample fEPSP waveforms recorded before and after HFS or LFS. **(C)** Time course plots of changes in the fEPSP slope expressed in percent of baseline. A stable increase in synaptic transmission efficacy (LTP) was observed in HFS-treated mice (*n* = 8) but not in mice receiving only LFS test-pulses; *n* = 4 **(D–I″)**. IHC images for Arc and c-Fos from bilaterally injected mice with AAV|sg9[+], mCherry, and Cas9 with the HFS (left) and Naïve (right) hippocampus visible. Scale bar in **(D)** is 500 μm while in **(E)** it is 100 μm. **(J)** Cell density box plot showing Arc and mCherry double-positive cells in the DG, ^*^statistically different (one way ANOVA, *p* ≤ 0.05, followed by Tukey’s HSD) (LFS *n* = 3, HFS *n* = 5). **(K)** Box plot displaying c-Fos immunoreactive cell density for LFS and HFS in the DG. LFS *n* = 3, HFS *n* = 5. **(L)** Correlation plot between mCherry+ and Arc+ cells in the dentate gyrus. Each animal sample is labeled by hemisphere (contralateral or ipsilateral) and group (LFS or HFS).

In one of the hemispheres, a bipolar stimulation electrode (NE-200, 0.5 mm tip separation, Rhodes Medical Instruments, Woodland Hills, CA) was inserted 3.9 mm posterior to bregma, 2.5 mm lateral to the midline, and 1.5 mm from the brain surface to target the angular bundle of the perforant path fibers. The insulated tungsten recording electrode (0.075 mm; A-M Systems #7960) was positioned ipsilaterally at 2 mm caudal to bregma and 1.5 mm lateral to the midline. The electrode was then lowered to the DG hilus (1.5–1.8 mm from the brain surface) with 0.1 mm increments while monitoring the response waveform profile evoked by a 400 μA test pulse (Figure 6B).

Electrode positioning was limited to a single penetration while maximizing the field extracellular postsynaptic potential (fEPSP) response (Figures 6A,A′). Baseline and post-HFS evoked responses were monitored using low-frequency stimulation (LFS) test pulses (pulse-width 0.1 ms, at 0.033 Hz). A 20 min period of baseline recording was obtained before the application of HFS. HFS was delivered at an intensity that produced a population spike of 30% maximum. The HFS protocol consisted of four trains of stimuli applied with an interval of 10 s; each train had 15 pulses at 200 Hz pulse width 0.1 ms. The stimulus intensity used for HFS was twice that used for test pulses. Evoked responses were recorded for 180 min after HFS (Figure 6C). A control group of mice received LFS test pulses but not HFS. Changes in the fEPSP slope were expressed as percent of baseline. After the recordings were completed, the electrodes were removed. The animals were immediately sacrificed, and the brain was prepared for immunohistochemistry (IHC), as detailed below.

### Tissue processing

Unstimulated mice were also used for IHC 3 weeks post-injection. The mice were first deeply anesthetized with sodium pentobarbital overdose (Apoteksbolaget, Sweden) and then transcardially perfused with 15 mL of the physiological saline solution followed by 100 mL of 4% paraformaldehyde (PFA). Brain tissue was extracted and post-fixed for 24 h at 4°C and subsequently stored. Using a sliding microtome, the brains were cut into 35 μm coronal sections. The sections were collected as one in six series and stored in an antifreeze solution (0.5 M sodium phosphate buffer, 30% glycerol, and 30% ethylene glycol) at −20°C until further processing. For DNA extraction, animals were euthanized using CO_2_, and the brains were extracted and rinsed from blood in an ice-cold saline solution. The whole brain was embedded in an optical cutting temperature (OCT) compound and snap-frozen in isopentane at approximately −75°C. Samples were then stored at −80°C until further processing.

### Molecular analysis

Brains used for molecular analysis were processed first by cutting away coronal sections using a cryostat. When approaching the AAVs deposits, the area was isolated using standard biopsy punches (Thermo Fisher Scientific). Genomic DNA was extracted from the dissected tissue using QIAamp DNA Micro Kit (Qiagen) and stored at −20°C. PCRs with primers targeting either the 5′ or the 3′ region of the insert were performed using Phusion Green hot Start II High Fidelity PCR Master Mix (Thermo Fisher Scientific). Primers were designed using Primer BLAST, a web-based tool from NIH (NCBI, 2019) (Supplementary Primer Table). For Sanger sequencing analysis, PCR amplicons were separated from the agarose gel using the Zymoclean Gel DNA Recovery Kit (Zymo research), purified, and shipped to Eurofins Genomics according to their instructions. For Next Generation Sequencing, amplicons were amplified using primers containing Illumina overhangs (Supplementary Primer Table) and indexed with Illumina Indexing primers. Libraries were then sequenced using a NextSeq 500 System.

### Bioinformatics analysis

For each sample, the Illumina sequencer-generated base call files were transformed into FASTQ format using bcl2fastq and demultiplexed. A Python script was generated which imports the FASTQ files and performs initial preprocessing. Preprocessing included filtering for real amplicons using BBduk as well as the removal of the primers using Cutadapt and subsequent clustering using Starcode (Martin, 2011; Zorita et al., 2015; Bushnell et al., 2017). Starcode clusters similar sequences based on sequence pair searches within a given Leveinshtein distance, which measures the difference between given sequences (Zorita et al., 2015). Starcode outputs the sequence clusters with the respective number of sequences found in each cluster.

### Alignments

By aligning the sequence clusters with relevant statistics against the reference sequence in a pairwise manner, we could determine the integrity of the sequence structures, the presence of potential scar sites caused by CRISPR/Cas9 technology, PCR-related issues, and the relative frequency of them. Pairwise alignment was used, where two sequences are compared to find the optimal alignment *via* a scoring system based on matches and mismatches. Furthermore, as potential gaps need to be introduced to find the optimal alignment, gap opening penalty (GOP) and gap extension penalty (GEP) were applied. GOP score applies stringency to the number of gaps the algorithm can insert into the alignment, whereas GEP controls the length of the gaps. To find the optimal pairwise alignment between the reference template and each sequence cluster, Needleman–Wunsch and Smith–Waterman algorithms with different scoring systems were applied to find an optimal alignment for each sample. Alignment plots were generated using Mview alignment software.

Through the amino acid level alignments, we could further establish how many of the aligned sequences produced a functional protein. This is especially important if a scar site has been formed. If the scar causes a loss of nucleotides at a specific locus, but the “post-scar” nucleotides are well aligned, a functional protein can still be formed. Furthermore, it was important to determine whether a stop codon could have been formed at these sites. To this end, the nucleotide sequences were translated in the determined correct frame as well as in the incorrect frames.

The complete analysis pipeline and detailed instructions can be found at https://github.com/MNM-LU/Arc-HITI.

### Immunohistochemistry

Each immunohistochemistry protocol started with tissue sections washed three times in phosphate buffer saline (PBS) pH 7.4. Next, the sections were blocked for 1 h in the blocking solution: 2.5% serum species where the secondary antibody was grown, 0.25% of Triton X-100, in PBS. Then, brain slices were incubated with primary antibody at room temperature (RT) overnight. On the second day, the primary antibody was washed away with PBS three times following incubation for 1 h in the blocking solution. Depending on the manufacturer’s instructions, the secondary antibody was diluted in 2.5% blocking solution, added to the sections, and incubated for 2 h at RT. This was followed by one PBS wash with DAPI 1 μg/mL and two without, to stain for nuclei. Sections were mounted on gelatin-coated glass slides and covered with PVA-DABCO mounting medium for confocal microscopy.

### Proximity ligation assay

NaveniFlex MR PLA kit was used to investigate protein–protein interaction and spatial localization for mCherry-Arc, mCherry-Stargazin, and mCherry-Bassoon (Klaesson et al., 2018). The primary antibodies used for PLA experiments were: mCherry (rabbit), and depending on the protein, we wanted to investigate the interaction with: Arc (mouse), stargazin (mouse), and bassoon (mouse). The primary antibody for mCherry (chicken) was always included for common IHC to localize the edited neurons. Free-floating tissue sections were washed three times with TBS-T in a small glass bottle to wash out antifreeze residual. Slides were incubated in antigen retrieval solution, Tris-EDTA pH 8.0 at 80°C for half an hour.

The tissue was then cooled down at RT and washed three times with TBS-T. Each sample was incubated 1 h at 37°C in 1X Blocking buffer (NaveniFlex MR PLA kit) and retained in free-floating suspension throughout the PLA reaction. Next, primary antibodies were diluted in Primary Antibody Diluent (NaveniFlex MR PLA kit) and incubated overnight at 37°C. The following day, the slides were washed in TBS-T three times under gentle agitation. Samples were then incubated for 1 h at 37°C with Probe 1 and Probe 2 diluted in Probe Diluent (NaveniFlex MR PLA kit). After three rounds of washing with TBS-T, slides were incubated with Reaction A and Buffer A (NaveniFlex MR PLA kit) for 1 h at 37°C. Following an additional three rounds of washing with TBS-T, slides were incubated with Reaction B and Buffer B for 30 min at 37°C. Following three more rounds of TBS-T washes, tissue was incubated in Reaction C and Buffer C for 1.5 h at 37°C. To localize successful knocked-in mCherry-Arc neurons, we performed one round of TBS-T wash followed by Cy3 anti-chicken antibody incubation for 1 h at RT. Incubation for 5 min with 1 μg/mL DAPI in TBS-T followed by two rounds of TBS-T washes was the last step before mounting the sections onto coated glass slides and covering them with cover slips.

The NaveniFlex protocol kit recommends having as a control a treated sample where the protocol is conducted as usual, but where one of the primary antibodies necessary to form the rolling circle amplification (RCA) puncta is omitted (see Table 3).

**Table 3:** Antibody information.

	Host	Company	Cat. Nr.	Dilution
Antibody (for IHC)				
GFP	Chicken	Abcam	Ab13970	1:10000
mCherry	Chicken	Abcam	Ab205402	1:1000
Arc	Rabbit	Synaptic System	156003	1:1000
Cy3 anti-chicken	Goat	Jackson	703–165-155	1:400
Alexa fluor 647 anti-mouse	Goat	Invitrogen	a21236	1:400
Alexa fluor 647 anti-rabbit	Goat	Invitrogen	a21245	1:400
Alexa fluor 647 anti-goat	Donkey	Invitrogen	a-21447	1:400
Antibody (for PLA)				
mCherry	Chicken	Abcam	AB13970	1:5000
mCherry	Goat	LSBio	LS-C204207	1:2500
Arc	Mouse	Santa Cruz	sc-17839	1:2000
Stargazin	Mouse	Abcam	Ab167445	1:2000
Bassoon	Mouse	Thermofisher	a21447	1:2000
Bassoon	Rabbit	Abcam	ab110426	1:2000

### *In situ* RCA for Cas9 using the BARseq2 protocol

The *in situ* RCA protocol was executed as described by Sun et al. (2021). Briefly, brain tissue was flash-frozen and embedded in an OCT compound. 10 μm sections were taken at the injection site and mounted on plus slides (Fisherbrand). The sections were fixed with 3.6%–4% PFA (Sigma-Aldrich) for 30 min and then washed with PBS (Thermo Fisher Scientific) for 5 min. Afterward, a HybriWell-FL chamber was installed, the sections were dehydrated in an alcohol series and then washed in 99.6% ethanol for 1 h at 4°C. Following, cDNA synthesis was performed with the following mix: primer concentration of 0.5 μm per mRNA, 1 U/µL RiboLock RNase inhibitor (Thermo Fisher Scientific), 0.2 μg/µL BSA, 500 μm dATP, dGTP, dTTP and 125 μm dCTP (Thermo Fisher Scientific), 100 μm Cy3-dCTP (Cytiva), 20 U/µL RevertAid H-Minus M-MuLV reverse transcriptase (Thermo Fisher Scientific) in 1× RT buffer was added to the reaction at 37°C overnight. cDNA was then crosslinked for 1 h with 50 mm BS(PEG)9 (Thermo Fisher Scientific) at room temperature.

Crosslinker was then neutralized with 1 M Tris–HCl at pH 8.0 for 30 min, and the sample was then washed with PBS-T twice. Padlock mix (1 U/µL RiboLock RNase Inhibitor (Thermo Fisher Scientific), 20% formamide (Thermo Fisher Scientific), 50 mM KCl, 0.4 U/µL RNase H (Qiagen) and 0.5 U/µL Ampligase (Epicentre) in 1× Ampligase buffer) was then added to the sample for 30 min at 37°C and 45 min at 45°C, and following a PBS-T wash, the sample was incubated with the rolling circle amplification mix (125 μM amino-allyl dUTP (Thermo Fisher Scientific), 0.2 μg/µL BSA (New England BioLabs), 250 μm dNTPs (Thermo Fisher Scientific), 5% glycerol and 1 U/µL ϕ29 DNA polymerase (Thermo Fisher Scientific) in 1× ϕ29 DNA polymerase buffer) overnight at room temperature.

After rolling circle amplification, the sample was crosslinked identically to post-cDNA synthesis. The samples were washed twice with hybridization buffer (10% formamide in 2× SSC mix), following which, a mix containing fluorophore-labeled probes (IDT) complementary to padlock probes’ products in hybridization buffer was added to the reaction for 10 min at room temperature. Fluorophores used were Alexa Fluor 488, Cy5, and Texas Red, conjugated to 5′ of probes. Finally, samples were washed in hybridization buffer twice with 3 min/wash, rinsed with PBS-T twice, and imaged.

### Laser scanning confocal microscopy

All immunohistochemistry analyses were performed using a Leica SP8 microscope. Images were captured using a HyD detector and always with the lasers set to be activated in sequential mode to avoid serial excitation. Solid-state lasers at 405, 448, 552, and 650 nm wavelengths were used to excite their respective fluorophores. A pinhole of 1AU was always retained during image acquisition. Leica objectives 5X/0.15, 20X/0.75, and 63X/1.40 were used during imaging acquisition. Multi-field imaging was normalized using the BaSiC ImageJ plugin and stitched using the MIST plugin (Chalfoun et al., 2017; Peng et al., 2017). Surface render images were created using a 3D module in the SP8 Leica software from z-stack images acquired with a 63X objective at a resolution of 1024 × 1024 pixel size.

Sections from *in situ* RCA experiments were imaged *via* HybriWell on a Nikon Eclipse Ti2 Inverted Microscope with a Nikon objective 20X/0.7.

### Artificial intelligence fluorescent cell, PLA puncta, and tissue volume quantification

The fluorescent cells were counted using an automated convolutional neural networks (CNN) algorithm in Aiforia Create on Aiforia’s cloud-based platform (Aiforia Technologies Oyj, Finland). This computer-assisted cell counting method is based on supervised learning as previously described (Penttinen et al., 2018).

The stained sections were digitized using a Leica SP8 confocal microscope at a resolution of 2048×2048 pixels when acquired with the 5X objective and at 1024 × 1024 pixels when using the 20X and the 40X objective. A total of 3 sections were acquired at an interval of 210 μm for all the animals. The digitized images were uploaded to Aiforia Hub (Aiforia Technologies Oyj, Finland). The number of positively labeled objects within the regions of interest was analyzed using the CNN algorithm trained to recognize either the cell bodies or the PLA puncta from the digital images (Supplementary Figure S1).

The algorithm consisted of two layers: the first layer segmented the brain tissue using semantic segmentation. In contrast, the second layer counted all the cells or PLA puncta using object detection in Aiforia Create. The brain tissue semantic segmentation was trained using the complexity of ‘complex’ with a field of view of 50 μm, whereas the object detection was trained using a complexity of “very complex”. The Aiforia version 5.3 was used for this study.

## Results

### Template strand HITI gene editing in the Arc 5′ UTR yields a high frequency of out-of-frame insertions

The 5′ UTR and the beginning of the Arc ORF were chosen to knock in the reporter sequence. The fusion of a tag at the N-terminal appeared to retain the capsid formation and neuronal uptake (Pastuzyn et al., 2018). This also leaves the 3′ UTR unperturbed, which is essential for Arc mRNA transfer to the dendritic processes for local translation into protein during synaptic plasticity (Steward et al., 2014).

AAVs were designed to knock in GFP or mCherry in-frame with the N-terminal of the Arc protein by targeting the template strand at the 2[−] position (Figure 2A). When injected into the mouse striatum and hippocampus, we observed sparse green fluorescent cells with neuronal morphology in the striatum and hippocampus of mice injected with GFP AAVs sg2[−] + Cas9 (Figures 2C,C′). No fluorescent cells were observed when the sgRNA was replaced with one targeting the Luciferase gene (sgLuc) (Supplementary Figures S1B,B′).

Insertion of mCherry in the Arc 5′ UTR using the same sg2[−] sgRNA resulted in red fluorescent neurons in the striatum and hippocampus (Figures 2D,D′), which was absent when injecting the corresponding control virus (AAV-mCherry sgLuc, Figures 2E,E′). To assess the transduction, we visualized the Cas9 mRNA using *in situ* rolling circle amplification (RCA, Supplementary Figure S1D). Editing efficiency was quantified through fluorescent cell density measurement in the transduced striatum and hippocampus. We found 313 ± 50 (mean ± SEM) labeled neurons in a volume of 1.45 ± 0.22 mm^3^ on average for both tissues. The mCherry AAV sg2[−] + Cas9 injection in the hippocampus yielded significantly higher transduction density than the GFP AAVs sg2[−] (one way ANOVA *p* < 0.001 followed by Tukey HSD *p* = 0.03), whereas there was no significant difference between the Arc sg2[−] active groups. All active constructs yielded higher density than the sgLuc groups (Figure 2F).

We extracted DNA from injected striatum and hippocampus (*n* = 3/group) and performed PCR with primers targeting either the 5′ or the 3′ region of the insert paired with Arc primers. We detected the expected band in every mouse injected with GFP or mCherry AAVs sg2[−] in the hippocampus and the striatum, whereas the PCR for the sgLuc negative control yielded no bands (Figure 2G; Supplementary Figure S1C). When knocking in mCherry, we observed by Sanger sequencing that the 5′ region of the insertion was aligned as expected, which was corroborated by next generation sequencing (NGS), where we found that 90% of the sequences matched with the expected outcome (Figures 3A,B; Supplementary Figures S2A-C). However, we found a deletion on the 3′ region of the insertion (Figure 3B), which resulted in an out-of-frame translation of the Arc ORF. In NGS, 67.9% of all edits resulted in a 2+ frameshift, whereas only 0.001% of the reads resulted in the correct sequence (Figure 3C). We concluded that while the editing and insertion efficiency is high at the sg2[−] position of the Arc 5′ UTR, the frameshift mutations resulted in a fluorescent protein not fused to the Arc protein. When knocking in GFP, we found similar but generally even worse results (Supplementary Figures S2A-C).

In parallel, we designed a second set of AAVs, inserting a myc-tag at the N-terminal of Arc using the sg4[−] target. However, we could not detect any tagged Arc in the striatum or in the hippocampus. Moreover, targeted PCR did not reveal an insertion of the tag in the expected insertion site.

### Targeting the Arc ORF 5′ region yields an in-frame mCherry knock-in

Since the mCherry tag was more robustly detected than the GFP tag, we decided to continue our studies with the mCherry reporter. We then set out to test if targeting the (+) strand instead of the (−) strand by HITI would yield a higher knock-in accuracy as the microhomology sequence at the scar site becomes shorter in the vulnerable 3′ edit. We designed AAVs targeting the coding strand on the Arc 5′ UTR sg1[+] and a region in the Arc ORF 9[+] (Figure 4B) and injected as previously in the striatum and hippocampus (Figure 4A).

Both approaches yielded a sparse number of fluorescent cells, 226 ± 53 mCherry+ cells in a volume of 1.03 ± 0.18 mm^3^ brain tissue (Figures 4C–D′), whereas a sgLuc control did not yield any fluorescent cells (Figures 4E,E′). The number of sg1[+] mCherry+ cells was significantly higher than in the striatum of sg9[+] transduced animals (one way ANOVA *p* < 0.001, followed by Tukey HSD *p* = 0.016) (Figure 4F). The PCR amplification targeting the 5′ and 3′ regions of the insert yielded expected bands for the approaches targeting Arc but not for the AAVs coding for a sgLuc control (Figure 4G). Unexpectedly, the sg1[+] approach resulted in a high frequency of frameshift induced at the 3′ edit site observed both by Sanger sequencing and NGS (Supplementary Figure S2B). The sg9[+] insertion, on the other hand, resulted in a high frequency of accurate edits both on the 5′ and 3′ region (Figures 5A,C), which translated to the expected in-frame insertion (Figures 5B,D). Hence, we concluded that the sg9[+] yielded a correct mCherry knock-in upstream and in-frame to the mouse Arc ORF and we continued our studies with this combination of AAVs.

In parallel, we revised the vectors based on the sg2[−] sgRNA to utilize open arm homology directed recombination, SATI (Suzuki et al., 2019) at the 3′ end. This could potentially prevent the micro-deletion observed above. The mCherry AAVs targeting the template strand sg2[−] were modified with a 3′ homology arm to utilize oaHDR at the mCherry-Arc junction and retained HITI at the 5′ end. We did not detect any fluorescent cells using this approach, despite a majority of correct insertions at the 5′ end (Supplementary Figure S4).

To further explore the potential mechanisms behind the microdeletions occurring with sgRNA2[−] and the insertions occurring with the sgRNA1[+] we processed the target sequences using two machine learning based tools: Lindel (Chen et al., 2019) and FORECasT (Allen et al., 2018). Both Lindel and FORECasT models very accurately predicted the insertions observed at the 3′ end of the sgRNA1[+] HITI approach but the Lindel model ultimately failed to predict the deletions observed at the 3′ end of the sgRNA2[−] HITI approach. FORECasT, on the other hand, correctly identified the sgRNA9[+] to give rise to significantly less NHEJ than the other two sgRNAs (Supplementary Figure S5). Thus, we conclude that FORECasT may be a valuable additional tool in the selection process for sgRNA targets used in HITI.

### Imaging of mCherry-Arc fusion protein after LTP induction

To study the expression of mCherry-Arc fusion protein in transduced neurons after induction of LTP, we unilaterally performed electrical stimulation of the perforant path and recorded field excitatory postsynaptic potentials (fEPSPs) in the DG hilar region. All mice were bilaterally injected with AAV|sg9[+], mCherry in the hippocampus (Figures 6A-A″). One group received high-frequency stimulation (HFS) to induce LTP along with low-frequency stimulation (LFS) test pulses, while a control group received LFS only (Patil et al., 2023) (Figures 6A-C). We observed a strong induction of mCherry fluorescence coupled with highly specific Arc induction on the HFS-stimulated DG (Figures 6D–F″; Supplementary Figure S6A).

The immediate early gene (IEG) c-Fos was equally induced in the ipsilateral DG (Figures 6G–I″; Supplementary Figure S6B). The HFS yielded significantly more Arc+/mCherry+ double positive granule cells on the stimulated side compared to the contralateral side (one way ANOVA followed by Tukey HSD *p* < 0.01). We also observed an increased number of Arc+/mCherry+ neurons in the DG after LFS and in the contralateral DG of HFS-treated animals compared to the contralateral DG of LFS-treated animals, albeit at much lower levels (one way ANOVA followed by Tukey HSD *p* < 0.05) (Figure 6J). To assess the difference in induction upon the two stimulation paradigms, we quantified the number of c-Fos + cells, where we observed a non-significant trend toward higher c-Fos + cell density in the HFS group (Figure 6K). Finally, the number of mCherry+ cells correlated significantly with both Arc+ and c-Fos + cells (Figure 6L; Supplementary Figure S7) (Pearson *R* 0.92 and 0.91, respectively, *p* < 0.001).

One important observation was the complete lack of mCherry positive neurons in the major input structure to the DG, the entorhinal cortex (giving rise to the perforant path), neither with or without LTP induction (Supplementary Figure S7).

### Assessment of chimeric protein formation using a proximity ligation assay

**Figure 7:**
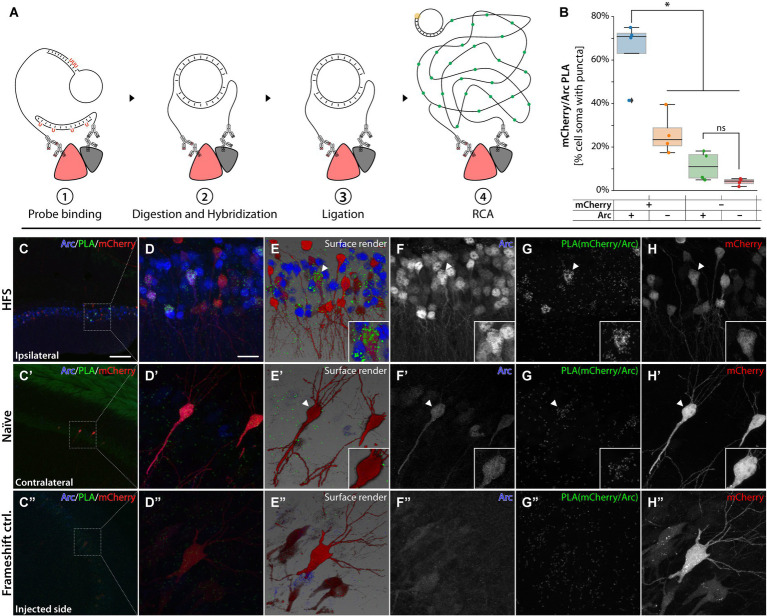
PLAs for Arc and mCherry. **(A)** Schematic illustration for the UnFold *in situ* PLA from Navinci technology. ① Species-specific antibodies paired with oligoprobes are added after the common primary antibody incubation. ② Digestion of the uracil residues frees the 5′ end, which hybridizes with the 3′ end oligoprobe sitting on the other antibody. ③ Ligation steps form a closed DNA strand. ④ A polymerase initiates an RCA that adds fluorescent nucleotides to be imaged later. **(B)** Box plot describing percentage of cells containing RCA puncta. “+” and “−” refers to staining positive or negative signal for mCherry and Arc protein. ^*^Statistically different (one way ANOVA, *p* ≤ 0.05, followed by Tukey’s HSD) (*n* = 3). **(B–H’)** PLA was conducted on an HFS animal where **(C′–H′)** is the contralateral, naïve hippocampus and **(C–H)** the ipsilateral, HFS-stimulated hippocampus. In **(C´–H´)** pyramidal neurons were imaged from CA1. In panel **(D–H)** granule cells from the DG. RCAs from PLA for Arc/mCherry are in green. Common IHC in red and blue to label mCherry and Arc cells, respectively. (**C″–H″)** PLA on CA3 from an animal injected with AAV| sg2[−], mCherry and Cas9. The scale bar in **(C)** is 100 μm, and in **(D)** it is 20 μm.

To evaluate whether the knock-in approach resulted in a chimeric mCherry-Arc protein, we performed a proximity ligation assay (PLA) using mCherry and Arc primary antibodies (Figure 7A) in both naïve (contralateral) and HFS stimulated (ipsilateral) hippocampus. Using machine learning based histological segmentation (Aiforia), we quantified the number of RCA puncta forming in HFS animals (*n* = 4). Importantly, we found significantly more puncta in the cell soma of mCherry+/Arc+ cells compared to single positive cells (Figure 7B, one way ANOVA followed by Tukey HSD *p* < 0.05) and mCherry-/Arc+ single positive cells did not have significantly more puncta than the negative control. While the RCA puncta were very abundant after HFS (Figures 7C–H), RCA signals were sparse in the naïve hemisphere and overlapped with the few Arc+/mCherry+ neurons primarily in CA1-3, suggesting the presence of focalized chimeric proteins (Figures 7C′−H′). On the ipsilateral hippocampus (HFS), we observed a large population of mCherry+ and Arc+ cells in the DG. Interestingly, although Arc+ and mCherry+ single-positive cells were observed, only double-positive DG cells generated an abundant RCA signal, indicating that HFS triggers enhanced expression of the mCherry-Arc chimeric protein (Figures 7D–H). To validate our findings, we performed PLA on brain sections from previously injected animals with the sg2[−] AAV system (Figure 2B). In this model, mCherry is expressed out-of-frame of the Arc ORF (Figures 2C,J). Importantly, we observed no RCA signal for these animals (Figures 7C″−H″), as expected when Arc and mCherry are expressed as independent proteins (Supplementary Figure S8). We did not observe any RCA in the negative control without Arc primary antibody (Supplementary Figure S9).

### The chimeric mCherry-Arc protein interacts with functional synaptic modules

**Figure 8:**
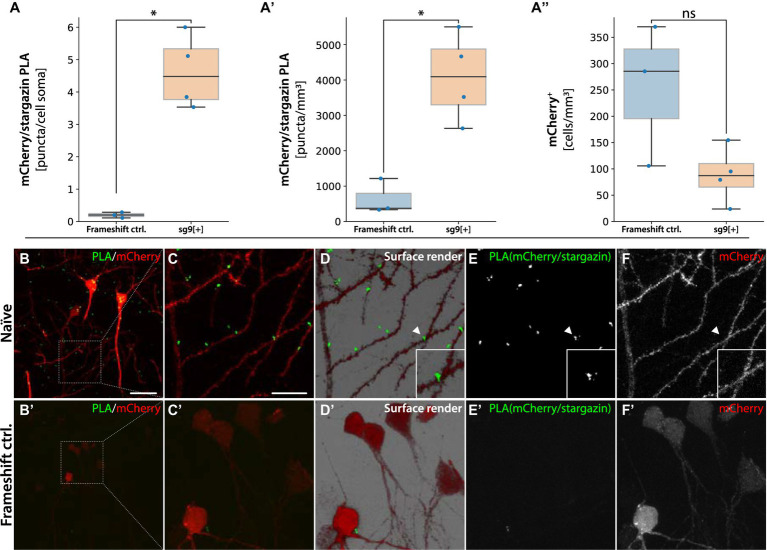
PLA for mCherry-Arc/Stargazin. In **(A)** quantification of RCA puncta per mCherry+ neuron in animal injected with sg2[−] causing the frameshift, and sg9[+] being in frame. In **(A′)** number of RCA puncta over the neuropil. In **(A″)** number of mCherry+ cells detected per mm^3^, ^*^statistically different, ns = not significant (one way ANOVA, *p* ≤ 0.05, followed by Tukey’s HSD), (*n* = 3). Naïve tissue, not HFS stimulated. In panel **(B)** pyramidal neurons from the hippocampal region CA1 of sg9[+] and frameshift control injected naïve tissue **(B′)**. Red mCherry labeled neurons contain green RCA puncta for mCherry interacting with stargazin. **(C–C′)** Magnified inset in the surface render panel **(D–D′)** showing mCherry-stargazin on a dendritic spine (arrowhead). **(E–E′)** RCA puncta signal coming from the protein–protein interaction of the mCherry-stargazin proteins. In panel **(F–F′)** signal coming from mCherry labeled cells. Scale bars in **(B)** and **(B′)** are 50 and 20 μm, respectively.

Arc has been shown to interact with several other proteins, including the postsynaptic partner stargazin (Zhang et al., 2015; Hallin et al., 2018). Stargazin is a transmembrane auxiliary subunit of AMPA-type glutamate receptors. We reasoned that a functional chimeric mCherry-Arc protein would bring the mCherry tag close enough to the stargazin protein to enable the formation of an RCA product after proximity ligation. When quantifying the mCherry/stargazin PLA in the sg9[+] injected hippocampus, we observed significantly more mCherry-Arc RCA puncta on mCherry+ neurons compared to the frameshift controls (animals injected with the sg2[−] the AAV system, Figure 8A). This effect was also seen when quantifying the RCA puncta on the neuropil (Figure 8A′). This difference was not due to more mCherry+ cells detected in the sg9[+] group compared to the frameshift controls (Figure 8A″). The mCherry/stargazin PLA resulted in RCA puncta primarily on dendritic spines (Figures 8B–F). Importantly, we did not observe any RCA puncta on the dendrites in the frameshift control (Figures 8B′−F′), nor when omitting the stargazin primary antibody (Supplementary Figure S9).

**Figure 9:**
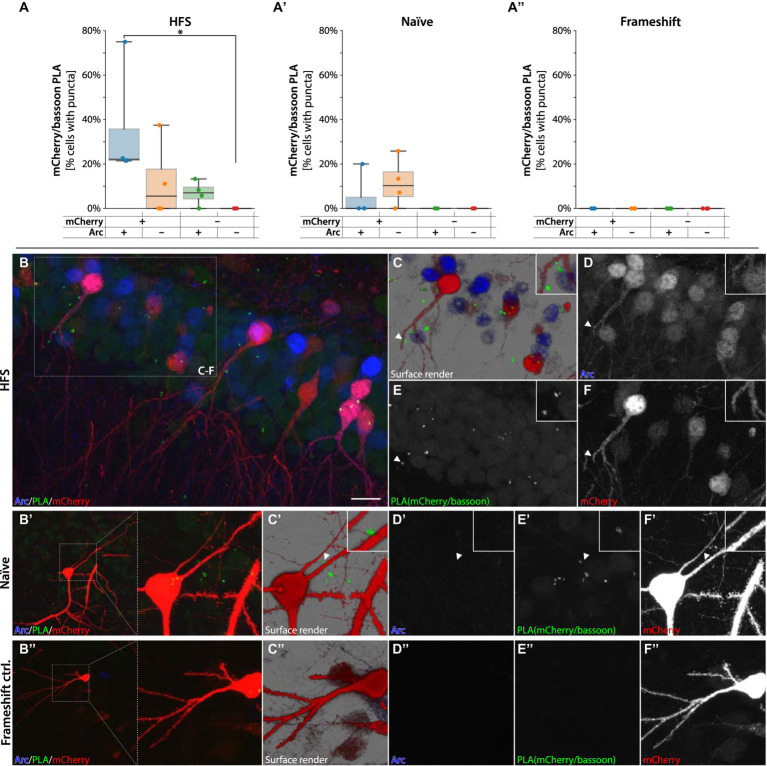
PLAs for mCherry-Arc with presynaptic protein bassoon. In **(A,A′)** quantification of RCA puncta from animals injected with sg9[+] in HFS and Naïve tissue, respectively. In **(A″)** quantification of RCA puncta from animals injected with sg2[−], causing the frameshift. ^*^Statistically different (one way ANOVA, *p* ≤ 0.05, followed by Tukey’s HSD). In panel **(B–F″)** images are acquired from the DG, fluorescent signals coming from granule cells. In red mCherry labeled neurons, in green RCAs for mCherry interacting with bassoon. In blue Arc positive cells. In panel **(C–C″)** surface render images with magnified insets showing mCherry-bassoon PLA adjacent to a granule cell dendrite. In panel **(D–D″)** signal coming from Arc positive cells. In panel **(E–E″)** RCA puncta coming from the protein–protein interaction of the mCherry-bassoon proteins. In panel **(F–F″)** signal coming from mCherry labeled cells. Scale bar in **(B)** is 20  μm.

A seminal report based on studies in cultured hippocampal neurons proposed that Arc protein capsids harboring Arc mRNA are transferred between neurons (Pastuzyn et al., 2018). However, evidence of Arc transfer between cells in the intact brain is lacking. As Arc is predominantly found in the postsynaptic neuronal compartment, e.g., in spines, we considered one route of Arc transfer could be from postsynaptic neuron to presynaptic terminal. To assess this possibility, we performed PLA of mCherry with bassoon, an abundant presynaptic scaffolding protein localized explicitly to the cytomatrix of the active zone in terminal boutons (Gundelfinger et al., 2015). If mCherry-Arc is transferred from spines to boutons, this might be detectable by PLA with bassoon. Performing PLA in the hippocampus of sg9[+] injected animals with primary antibodies against mCherry and bassoon revealed several RCA forming in close proximity to mCherry+ neurons, often near dendritic spines, suggesting that the chimeric protein transferred from its cell of origin (Figure 9). Quantification using Aiforia of the HFS animals revealed a significantly higher percentage of cells with RCA puncta in mCherry+/Arc+ cells compared to negative cells (one way ANOVA followed by Tukey HSD *p* < 0.05, Figure 9A). The mCherry/bassoon RCA puncta were found primarily adjacent to, but not on, dendrites (Figures 9B–F). In the Naïve animals, RCA puncta were found sparsely around mCherry+ cells (Figures 9A′−F′). Importantly, we observed no RCA signal from animals injected with the frameshift control (sg2[−], Figures 9A″−F″), nor when incubating only the bassoon primary antibody (Supplementary Figure S9). In summary, our data suggest that the sg9[+] HITI approach yielded a functional chimeric mCherry-Arc protein with pre- and postsynaptic activity and gives *in vivo* evidence favoring the inter-neuronal transfer of Arc hypothesis.

## Discussion

In this study, we aimed to tag the Arc protein *in situ* utilizing AAV-mediated CRISPR/Cas9 and the DNA self-repair mechanisms. This HITI approach was efficiently delivered using our engineered AAV capsid (MNM008) in the mouse striatum and hippocampus. Previous studies adopted the HITI system to knock in small tags in the 3′ region of their gene of interest (Gao et al., 2019; Willems et al., 2020). Here, we instead aimed for the 5′ region of the Arc gene to avoid possible disruption of regulatory elements involved in the mRNA splicing, micro-RNA regulation nonsense-mediated decay, dendritic transport, and metabolism (Giorgi et al., 2007; Wibrand et al., 2012).

Our initial experiments showed that the HITI system can lead to unpredictable results. Targeting the template strands [−] for the knock-in of a fluorescent reporter on the 5′ UTR by HITI yielded high fluorescence of sparsely distributed neurons. However, the NGS analysis revealed the mCherry or GFP coding sequence was mainly integrated out-of-frame respective to the Arc ORF. This was caused primarily by recurrent deletion of bases. While our sequence sample size is too small to find common patterns in the target sequences, such deletions may be caused by microhomology-mediated end joining (MMEJ) repair (McVey and Lee, 2008). The FORECasT machine learning model (Allen et al., 2018) successfully identify the best target sequence and predicted some of the insertions and deletions observed. With the increasing number of studies utilizing HITI and related technologies *in vivo,* it may be possible to refine this model and make it even more predictive for target selection.

The SATI-based approach resulted in our hands only in detectable integration on DNA but not protein levels. Our interpretation of this finding is that the SATI-based integrations may primarily occur in non-neuronal cells. As Arc expression is primarily restricted to neurons in the brain (all mCherry+ cells have a neuronal morphology in the functional HITI groups), such integration would not result in any fluorescent cells *in vivo*. This explanation is supported by the very low efficiency of the integration compared to the same sg2[−] HITI group and the fact that the Cas9 and sgRNA are expressed under ubiquitous promoters.

We achieved an in-frame mCherry-Arc sequence when targeting inside the 5′ region of the Arc ORF. In the unstimulated hippocampus, we observed sparse labeling with mCherry in the DG (89 ± 21 cells per mm^3^). This density suggests labeling of 30%–40% of the 250 Arc+ cells per mm^3^ reported under home-cage conditions in mice (Cleland et al., 2017). Scattered mCherry+ neurons were also found outside the DG in all transduced areas of the hippocampus (195 ± 85 cells per mm3) in line with fluorescence *in situ* hybridization showing low single percentage of Arc+ neurons in both CA1 and CA3 under home-cage conditions (Miyashita et al., 2009).

We observed that the majority of the mCherry+ cells were also Arc+. In line with this finding, we observed proximity ligation-based fluorescence for the antibody pair mCherry/Arc predominantly on mCherry/Arc double-fluorescent neurons. The NGS quantification of DNA edits would predict a near-universal accuracy of the knock-in, which is not supported by the protein observations *in situ*. It is possible that the integrated donor template (DT) sometimes contains the ITR region due to partial digestion of the AAV genome. ITR-containing donors are highly prone to insertion into the genome at double-strand breaks (Hanlon et al., 2019). Due to the very suppressive effect of the ITR structure on both PCR and NGS sequencing, we have not yet been able to devise an approach to detect such integrations, should they indeed occur.

There are three possible scenarios for ITR-driven integration using the HITI approach. The first is that both ITRs are retained (both upstream and downstream of mCherry). This would not result in any detectable mCherry, as the upstream ITR would block the transcription. The second scenario is that only the upstream ITR is retained, and this would have the same effect. The interesting scenario is the last one, where the upstream ITR is removed, but the downstream ITR is retained. There are good reasons to suspect that this may be the most prevalent form. Most likely due to the proximity of the sgRNA target site to the ITR in our constructs.

In this scenario the 5′ UTR of Arc would be linked to mCherry without hindrance, but the transcript would be terminated by the downstream ITR. Such integration events would result in mCherry+ cells driven by the Arc promoter. Indeed, we observed that total mCherry+ intensity increased dramatically after HFS both in Arc+ and Arc− neurons. Of note is that the mCherry/Arc PLA analysis revealed no signal in out-of-frame knock-in animals expressing an Arc promoter-driven mCherry reporter (i.e., not a chimeric protein). This discrepancy could be due to differences in the cutting efficiency of the CRISPR/Cas9 on the different target sites in the AAV DT with lower cutting efficiency resulting in more ITR containing DT to be inserted.

Induction of LTP by HFS resulted in a very strong induction of both Arc and mCherry in the ipsilateral DG. Surprisingly, we observed some but markedly less Arc and mCherry induction also in the contralateral DG after HFS and even less but detectable induction in the ipsilateral DG after LFS. While we have not been able to identify the cause of this, it is worth to note that regardless of the means of Arc induction, the level of mCherry fluorescence correlated strongly with both Arc and cFos (Figure 6L; Supplementary Figures S7D-E) in the DG, further supporting its functional integration into the Arc mRNA.

We continued our PLA studies in the in-frame knock-in mice. Here, mCherry interacting with synaptic proteins would provide support for a retained function of the chimeric mCherry-Arc protein. The observation of mCherry/stargazin proximity-ligated RCA products in mCherry+ dendrites at spine-like structures in CA1 pyramidal neurons supports that the translated chimeric protein is targeted correctly in the neuron and retains the interaction with AMPAR stargazin/TARPγ2 complexes in the postsynaptic membrane. AMPARs are tethered at synapses by binding of the TARP cytoplasmic tail to a postsynaptic scaffolding protein such as PSD95 (Bats et al., 2007). Arc also binds to the TARP cytoplasmic tail and might inhibit AMPAR tethering and increase the surface diffusion of receptors (Zhang et al., 2015; Zhang and Bramham, 2021).

Although Arc is generally considered to reside in the postsynaptic compartment rather than the presynaptic, some observations suggest that it can be presynaptic. Electron microscopy has indicated the existence of Arc in both boutons and glia (Rodriguez et al., 2005, 2008). Similarly, the presynaptic protein bassoon was identified by tandem affinity purification as one of 72 proteins interacting with the N-lobe ligand binding motif of Arc (Fernandez et al., 2017). Another observation fitting with these data is the transfer of *Drosophila* Arc seen in extracellular vesicles from the presynaptic bouton of motor neurons to the muscle in the *Drosophila* larvae (Ashley et al., 2018).

Strikingly, we also observed PLA signal when assessing the proximity between mCherry and bassoon. The fluorescent RCA products surround the mCherrry+ granule cells dendrites and soma without overlapping with the mCherry+ structures. In this regard, the pattern differs from the mCherry/Stargazin PLA, where the puncta overlap the mCherry positive spines. These data provide further support for that Arc can reside in the same presynaptic cellular compartment as bassoon. As presynaptic projections (perforant path terminals) show no signs of being directly transduced (i.e., not mCherry+, Supplementary Figure S7F), the fusion protein is most likely produced in the neighboring highly mCherry+ granule cell. This is supported by the finding that mCherry/bassoon RCA products form onto mCherry/Arc double positive granule cells in the DG but not onto single positive neurons as seen in Figure 9B. Thus, they do appear to follow the edited state and synaptic activity-induced expression of mCherry-Arc in the postsynaptic neuron and not the innervation pattern of the presynaptic neuron. There are reasons to assume that single antibody fluorescent detection would be more sensitive than PLA detection as the latter requires the successful binding of 4 antibodies compared to 2, proximity binding, and multiple enzymes for RCA formation (Klaesson et al., 2018). We also observe much broader fluorescence in the correctly edited cells (mCherry+/Arc+) than the PLAs toward the same fusion protein, further supporting this assessment. Once the RCA happens, however, the signal to background and specificity is better thanks to the massive signal amplification resulting from the RCA. The observed mCherry/bassoon PLAs thus favor the hypothesis that Arc has the capacity for inter-neuronal transport, as previously proposed based on *in vitro* studies (Pastuzyn et al., 2018). However, additional studies are needed to confirm the exact mechanism of this transfer and if any mRNA is indeed transported between the neurons *in vivo*.

## Data availability statement

The original contributions presented in the study are included in the article/Supplementary materials, further inquiries can be directed to the corresponding author.

## Ethics statement

The animal experiments were approved and performed under the Swedish Animal Welfare Agency regulations and in agreement with the local ethical committee for the use of laboratory animals (Ethical permit no. M 66-16 and 4111/2021-m).

Author contributions

MA, TB, and CB: conceptualization. MA, JP, TM, AR, and JR: formal analysis. MA, TB, JP, TM, and CB: methodology and writing—original draft. MA and TB: visualization. MA, TB, JP, TM, CB, MÅ, MD, and LQ: writing—review and editing. All authors contributed to the article and approved the submitted version.

## Funding

The research leading to these results has received funding from Horizon 2020 NSC Reconstruct (874758); Knut & Alice Wallenberg Foundation (KAW 2018-0040); Olle Engkvist foundation, The Mats Paulson foundation; Swedish Parkinson Foundation; Swedish Brain Foundation; Vinnova (2020-04702); Strategic Research Area at Lund University Multipark and the Segerfalk foundation. Work in the CB lab was supported by the Trond Mohn Foundation (TMS2021TMT04). TM was supported by a PhD fellowship from the University of Bergen.

## Conflict of interest

DK was employed by Aiforia Technologies Oyj.

The remaining authors declare that the research was conducted in the absence of any commercial or financial relationships that could be construed as a potential conflict of interest.

## Publisher’s note

All claims expressed in this article are solely those of the authors and do not necessarily represent those of their affiliated organizations, or those of the publisher, the editors and the reviewers. Any product that may be evaluated in this article, or claim that may be made by its manufacturer, is not guaranteed or endorsed by the publisher.
