# Supplementary material for: Visualizing Arc protein dynamics and localization in the mammalian brain using AAV-mediated in situ gene labeling
Source: Front Mol Neurosci. 2023 Jun 15;16:1140785. doi: 10.3389/fnmol.2023.1140785 (PMC10321715; doi:10.3389/fnmol.2023.1140785)
Supplement: Supplementary file 3 [file Image_1.pdf]

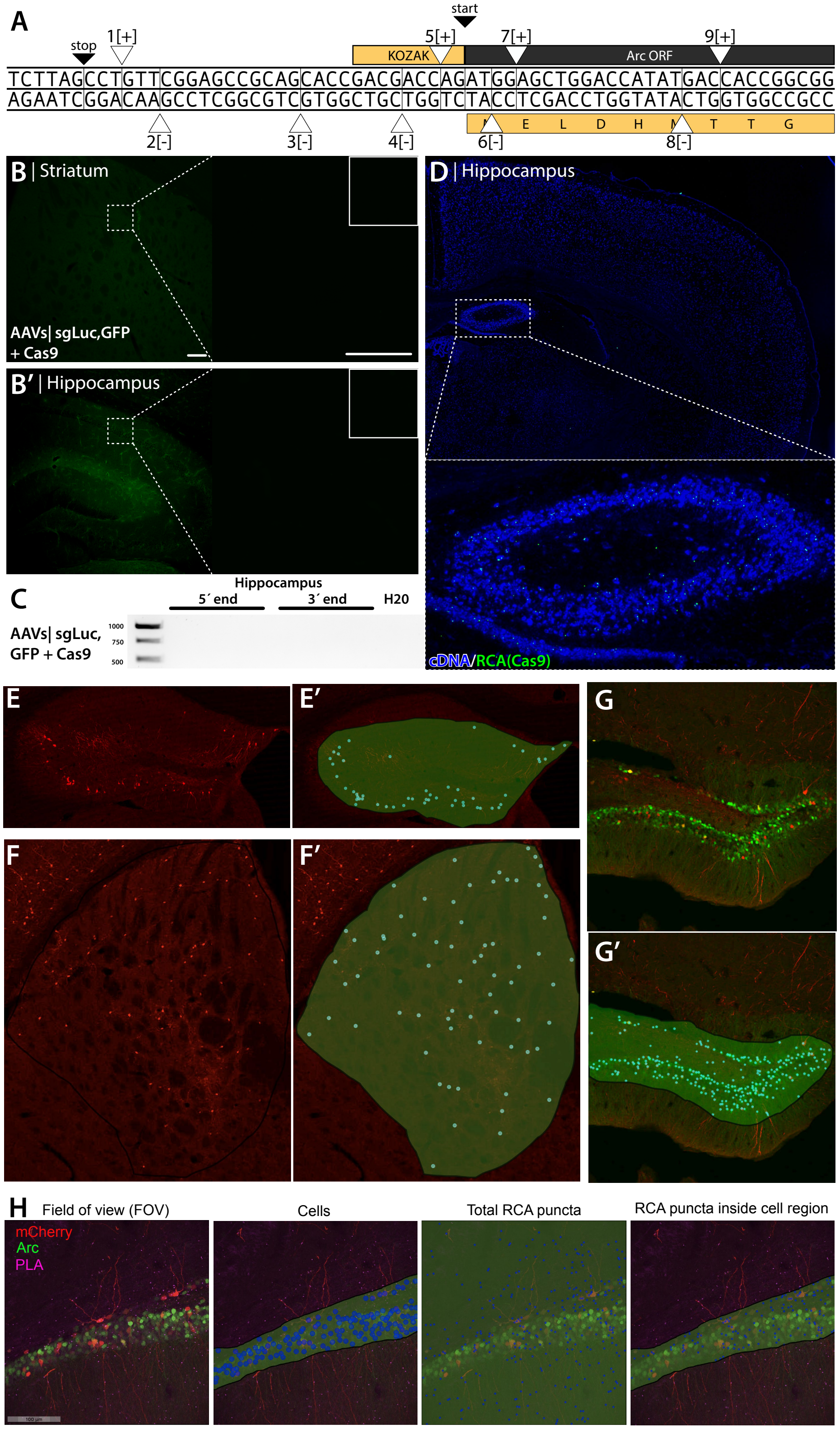

Supplementary Figure S1 | A. 5′ Arc sequence showing all possible insertion sites in the coding strand [+] and the template strand [-] based on the PAM sequence for spCas9. TAG stop codon in frame with the ATG start codon of the Arc gene. **B-B‴.** IHC images from striatum and hippocampus mouse brain from the control group for AAV| sgLuc,GFP. In B, the left scale bar is 50 µm, and in the right, it is 20 µm. **C.** Molecular analysis from the same control group. **D.** Section from the hippocampus of an animal injected with AAV | sg9[+], mCherry, and Cas9 where the in situ RCA using a BARseq2 protocol revealed in blue all transcripts and in green RCA specifically for Cas9. **E-G′** 5X images upload to Aiforia software for unbiased machine learning cell count. **H.** Exemplified PLA puncta detection on cells soma region and outside by Artificial Intelligence. The figure shows mCherry-bassoon PLA detection, but the same detection principle applies to every PLA.
