# Supplementary material for: Visualizing Arc protein dynamics and localization in the mammalian brain using AAV-mediated in situ gene labeling
Source: Front Mol Neurosci. 2023 Jun 15;16:1140785. doi: 10.3389/fnmol.2023.1140785 (PMC10321715; doi:10.3389/fnmol.2023.1140785)
Supplement: Supplementary file 4 [file Image_2.pdf]

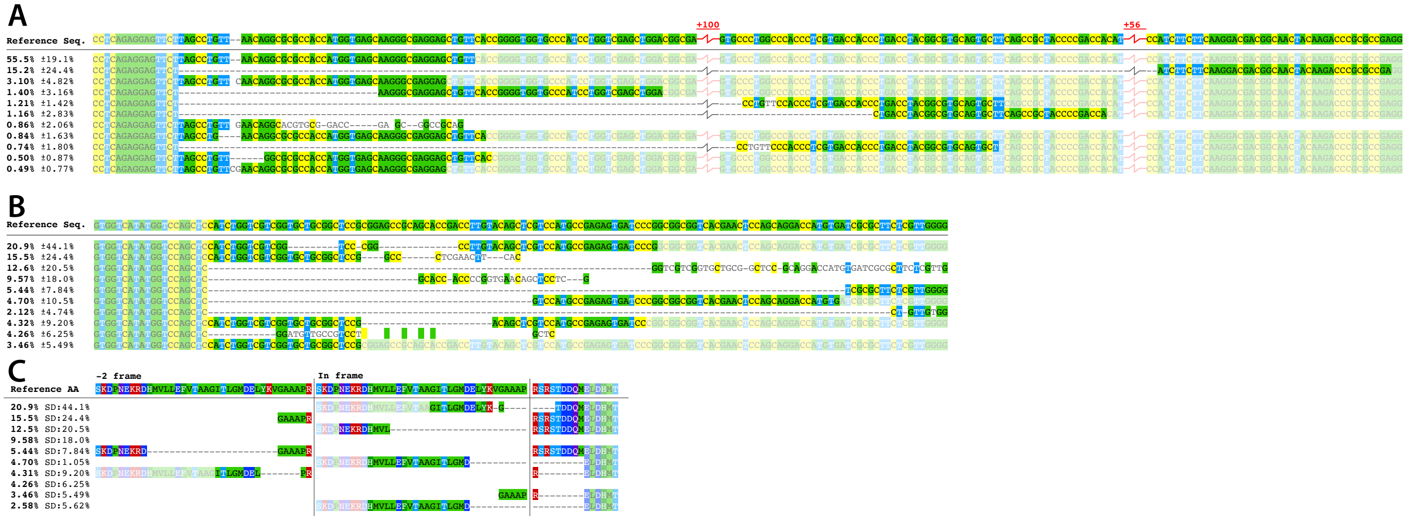

Supplementary Figure S2 | NGS analysis of HITI sg2[-] GFP. **A.** Sequence alignment for the GFP insert conducted on the 5' region. **B.** Sequence alignment for the GFP insert, conducted on the 3' region. **C.** Amino acid sequence of the 3' end region of GFP. Each amplicon is depicted showing the frameshift resulting from the deletion between the inserted sequence and the Arc gene.
