# Supplementary material for: Visualizing Arc protein dynamics and localization in the mammalian brain using AAV-mediated in situ gene labeling
Source: Front Mol Neurosci. 2023 Jun 15;16:1140785. doi: 10.3389/fnmol.2023.1140785 (PMC10321715; doi:10.3389/fnmol.2023.1140785)
Supplement: Supplementary file 6 [file Image_4.pdf]

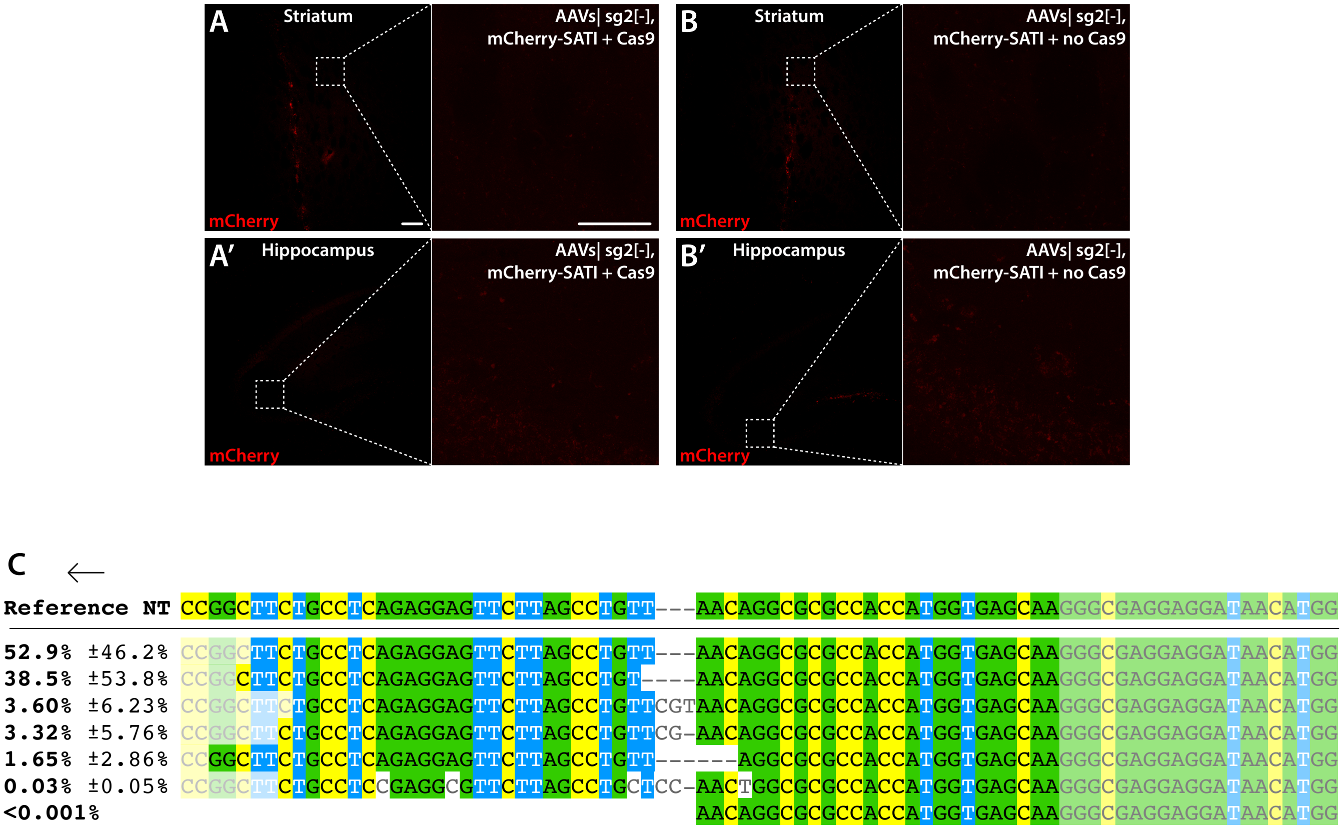

Supplementary Figure S4 | IHC and 5´analysis for AAV| sg2[-],mCherry-SATI. **A-A′.** IHC images from striatum and hippocampus mouse brain from animals injected with AAV| sg2[-],mCherry-SATI. In A, the left scale bar is 50 μm and in the right is 20 μm. **B-B′** and its respective control group injected without Cas9 **C.** Sequence alignment for the mCherry insert conducted on the 5′ region
