# Supplementary material for: Visualizing Arc protein dynamics and localization in the mammalian brain using AAV-mediated in situ gene labeling
Source: Front Mol Neurosci. 2023 Jun 15;16:1140785. doi: 10.3389/fnmol.2023.1140785 (PMC10321715; doi:10.3389/fnmol.2023.1140785)
Supplement: Supplementary file 7 [file Image_5.pdf]

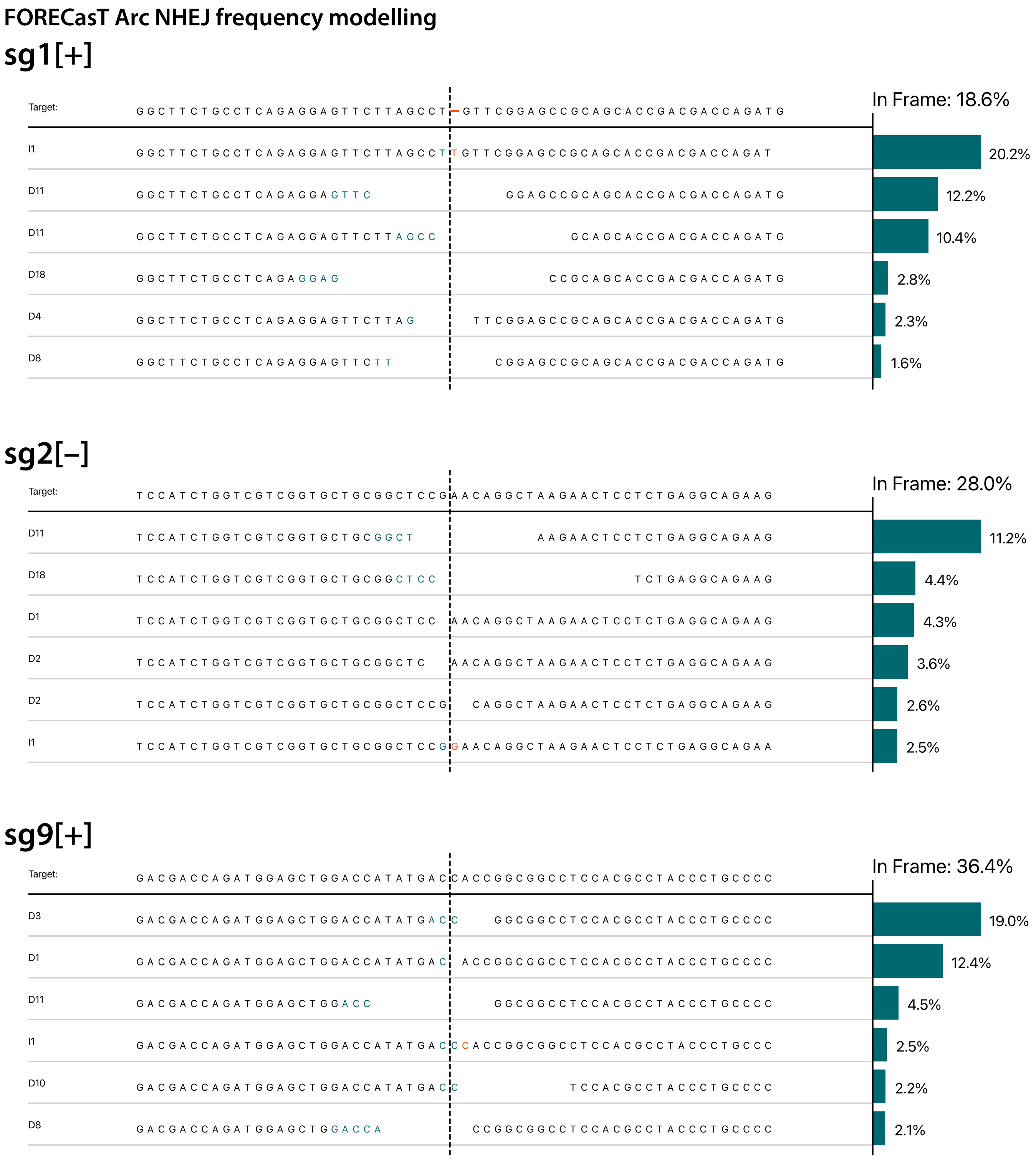

Supplementary Figure S5 | FORECasT Arc NHEJ frequency modeling. Percentage of most probable NHEJ events happening at the insertion sites for sg1[+], sg2[-], and sg9[+]. Below the target, D: deletion, I: insertion. Next to it are reported the number of nucleotides.

Dashed line = cut site, red = inserted nucleotides, green = microhomology (location ambiguous).
