# Supplementary material for: Visualizing Arc protein dynamics and localization in the mammalian brain using AAV-mediated in situ gene labeling
Source: Front Mol Neurosci. 2023 Jun 15;16:1140785. doi: 10.3389/fnmol.2023.1140785 (PMC10321715; doi:10.3389/fnmol.2023.1140785)
Supplement: Supplementary file 8 [file Image_6.pdf]

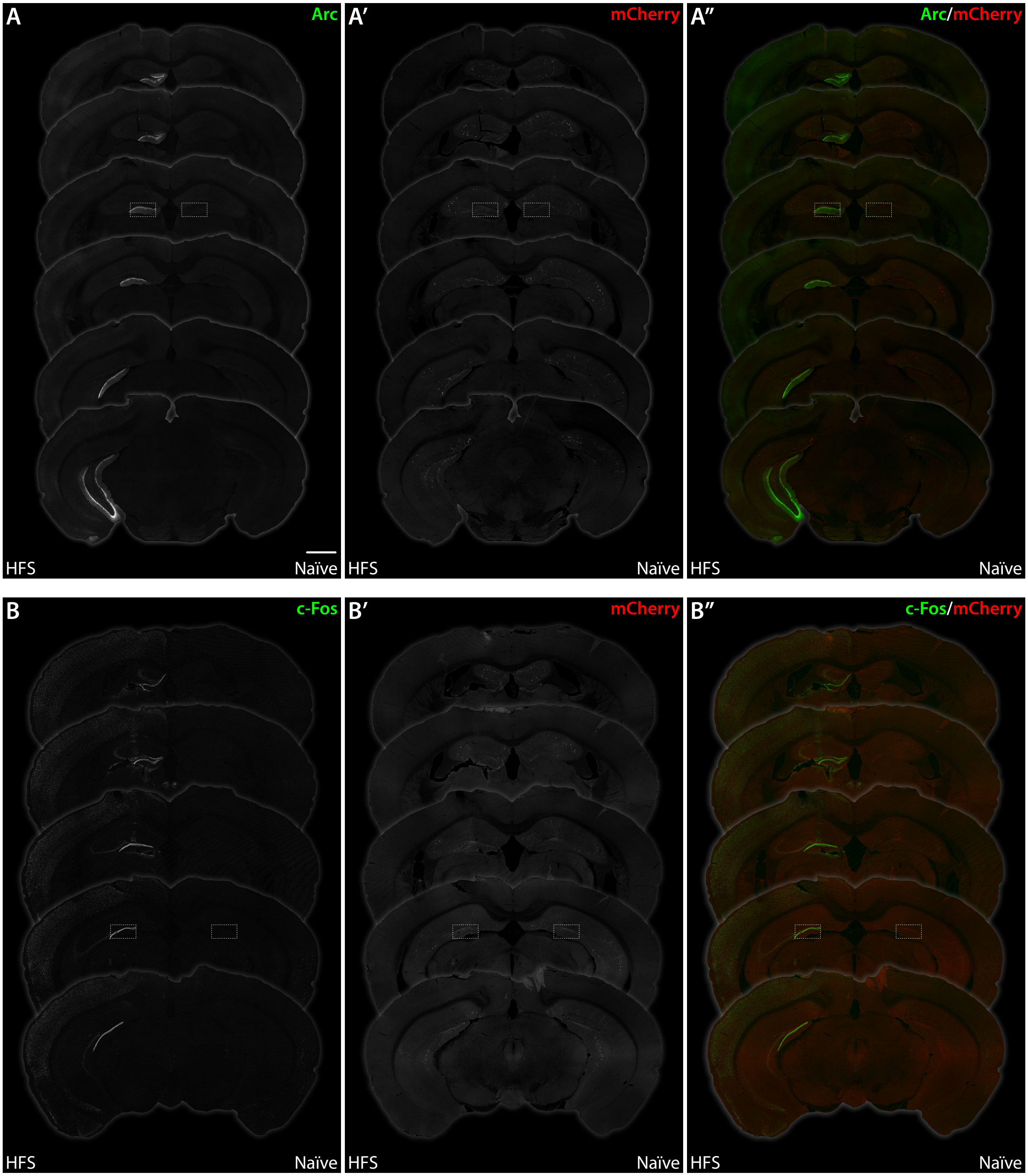


**Supplementary Figure S6** | IHC from HFS animal. Serial section rostral to caudal, top to bottom, respectively. The left hemisphere is the High-Frequency stimulation (HFS) side while the right hemisphere is the Naïve un-stimulated one **A-A″.** Double staining for Arc (in green) and mCherry (in red). **B-B″.** Double staining for c-Fos (in green) and mCherry (in red). In A, the scale bar is 1 mm.
