# Supplementary material for: Visualizing Arc protein dynamics and localization in the mammalian brain using AAV-mediated in situ gene labeling
Source: Front Mol Neurosci. 2023 Jun 15;16:1140785. doi: 10.3389/fnmol.2023.1140785 (PMC10321715; doi:10.3389/fnmol.2023.1140785)
Supplement: Supplementary file 9 [file Image_7.pdf]

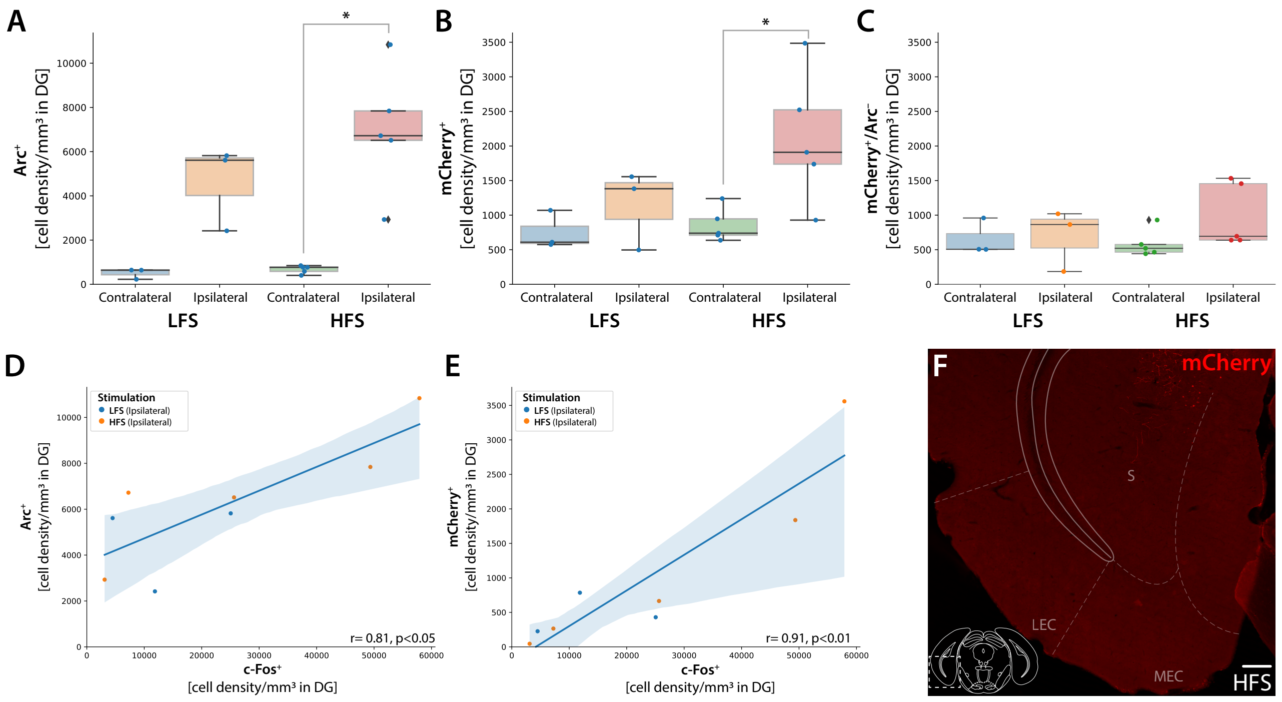

Supplementary Figure S7 | Cell count and correlation plot in HFS and LFS animals. **A.** Cell density box plot showing Arc+ cells in the DG, * p<0.05. **B.** Cell density box plot showing mCherry+ cells in the DG in LFS (n=3) and HFS group (n=3), * p<0.05. **C.** Cell density box plot showing mCherry+/Arc+ cells in the DG **D.** Correlation plot between Arc+ and c-Fos+ cells in the DG. **E.** Correlation plot between mCherry+ and c-Fos+ cells in the dentate gyrus. Each animal sample is labeled by hemisphere (contralateral or ipsilateral) and group (LFS or HFS). **F.** Confocal microscopy with IHC for mCherry of the entorhinal cortex after HFS. Abbreviations: LEC=lateral entorhinal cortex, MEC=medial entorhinal cortex, S=subiculum. Scale bar in F represents 200 µm.
