# Supplementary material for: Visualizing Arc protein dynamics and localization in the mammalian brain using AAV-mediated in situ gene labeling
Source: Front Mol Neurosci. 2023 Jun 15;16:1140785. doi: 10.3389/fnmol.2023.1140785 (PMC10321715; doi:10.3389/fnmol.2023.1140785)
Supplement: Supplementary file 10 [file Image_8.pdf]

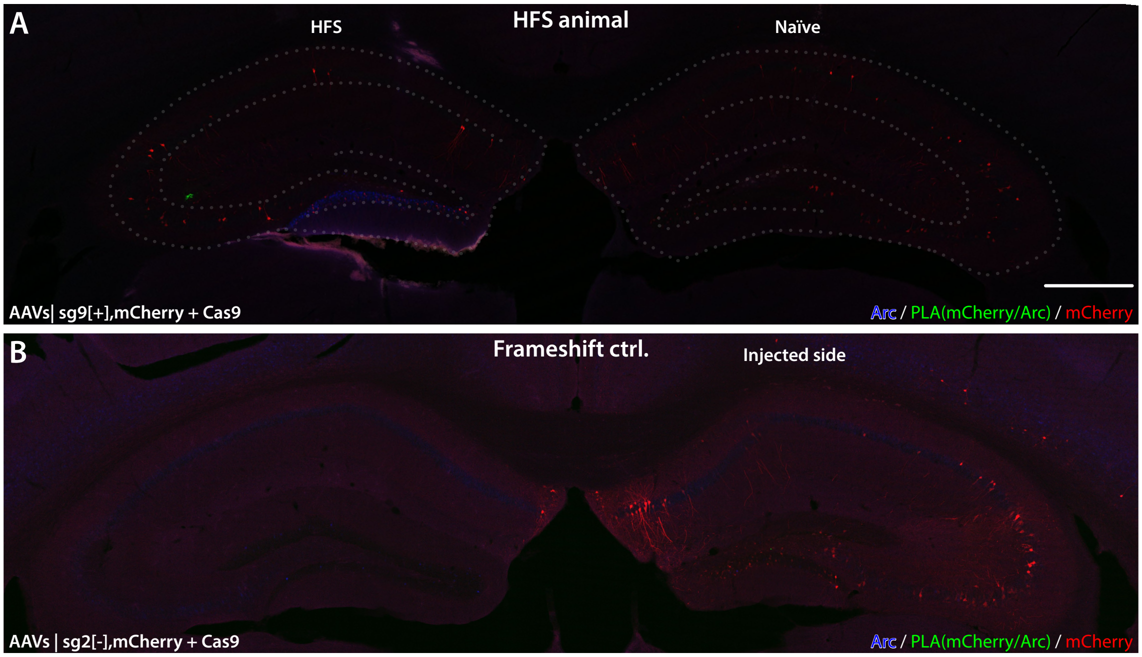

Supplementary Figure S8 | mCherry-Arc PLA on HFS, Naïve, and frameshift ctrl samples. **A.** Overview of the hippocampus, HFS animal. **B.** Overview of the hippocampus, from a frameshift animal injected with AAV| sg2[-],mCherry + Cas9. Scale bar 1 mm.
