# Supplementary material for: Visualizing Arc protein dynamics and localization in the mammalian brain using AAV-mediated in situ gene labeling
Source: Front Mol Neurosci. 2023 Jun 15;16:1140785. doi: 10.3389/fnmol.2023.1140785 (PMC10321715; doi:10.3389/fnmol.2023.1140785)
Supplement: Supplementary file 11 [file Image_9.pdf]

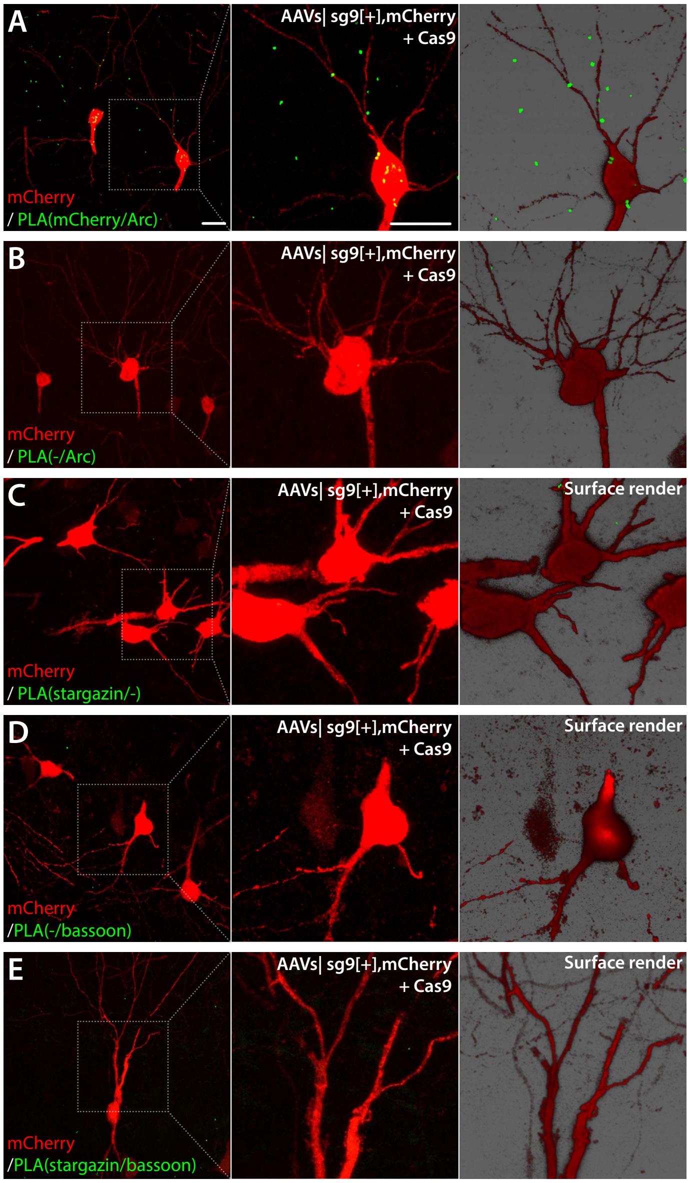

Supplementary Figure S9 | mCherry-Arc PLA in Naïve animals. In panel **A,** images containing neurons from hippocampal sections, where primary antibodies for the PLA protocol were included. In red mCherry labeled neurons, green RCA puncta from mCherry-Arc protein-protein interaction. In panels **B, C,** and **D,** experimental control where only one antibody was included during the PLA (Arc, stargazin, bassoon antibody, respectively) in panel **E,** PLA for Stargazin and Bassoon proteins interaction in green. Labeled in red, mCherry positive neuron. Scale bars in A are 20 µm.
